# Biogeography and Climate Drive Population Divergence and Genomic Vulnerability in High Altitude Endemic Bird

**DOI:** 10.1111/mec.70274

**Published:** 2026-02-21

**Authors:** Nan Wang, Prashant Ghimire, Pritam Chhetri, Nishma Dahal, Cheng Yi, Tong Zhang, Suonan Zhuoga, Zhaxi Jiangyong, Sangeet Lamichhaney

**Affiliations:** ^1^ School of Ecology and Nature Conservation Beijing Forestry University Beijing China; ^2^ Department of Biological Sciences Kent State University Kent USA; ^3^ Biotechnology Division CSIR‐Institute of Himalayan Bioresource Technology Palampur HP India; ^4^ Central South Inventory and Planning Institute of National Forestry and Grassland Administration Changsha China; ^5^ Forestry and Grassland Bureau of Yusu Yusu Qinghai China; ^6^ School of Biomedical Sciences Kent State University Kent Ohio USA

**Keywords:** biogeography, climate, high altitude, local adaptation, Sino‐Himalaya, Tibetan Partridge

## Abstract

High‐elevation systems support species adapted to extreme conditions, and their rugged terrain and variable microclimates strongly shape evolution and persistence. Yet few studies have evaluated how geography and climate jointly shape genetic diversity, local adaptation and vulnerability to environmental change. Here, we investigate these processes in the Tibetan Partridge ( *Perdix hodgsoniae* ), a high‐altitude endemic distributed across arid western and humid northeastern regions of the Sino‐Himalayan landscape. This region's complex topography and contrasting climatic conditions provide a natural setting for examining population divergence, climate‐associated adaptation and future resilience. We integrated whole‐genome sequencing, ecological, climatic, landscape and morphological data to examine current patterns of local adaptation and forecast climate‐induced risks. Our findings show that both biogeographic barriers and climatic gradients drive rapid population divergence in *P. hodgsoniae* , reflected in distinct morphological traits and population genetic structure. Populations in dry, fragmented western landscapes show adaptation to temperature, whereas those in humid northeastern regions exhibit adaptation primarily to precipitation. These contrasting adaptive trajectories lead to varying levels of vulnerability, with arid, isolated landscapes limiting gene flow and genetic diversity, thereby heightening sensitivity to future climate change. In contrast, humid regions maintain stronger connectivity and larger effective population sizes, supporting higher genetic diversity and facilitating precipitation‐linked adaptation. Together, we demonstrate that mountain landscapes function as a ‘double‐edged sword’ by simultaneously generating and limiting biodiversity through isolation, and by constraining persistence within microclimatic refugia. This study underscores the value of integrating genomic, ecological, climate and landscape data to uncover mechanisms of divergence and inform conservation planning under rapid environmental change.

## 1 Introduction

Mountains are biodiversity‐rich ecosystems shaped by complex geological processes, where steep geological and environmental gradients drive strong biological diversification. Although mountains only cover 25% of the landmass, they harbour nearly 87% of the world's terrestrial biodiversity (Colwell et al. 2008; Rahbek et al. 2019), making them critical yet highly climate‐vulnerable regions (Knight 2022). As the environment changes, many species shift upslope to track suitable conditions (Freeman et al. 2018; Rödder et al. 2021). However, species already occupying high elevations have limited opportunities for upward movement, and their dispersal is further constrained by rugged terrain and steep climatic gradients. These factors restrict their ability to track shifting climates and may increase their vulnerability to environmental changes (Sánchez‐Montes et al. 2018; Urban 2018). In this context, several critical questions arise: How do such species persist in geographically complex regions characterised by fragmented habitats and highly variable environmental conditions? Are their responses to environmental change consistent across their range, or do local environmental factors drive divergent adaptive strategies? Furthermore, do some populations exhibit greater vulnerability due to differences in biogeographic constraints or genomic architecture?

Addressing these questions related to their current and future persistence requires a comprehensive analysis of local populations of high‐altitude endemic species across their full distributional range, encompassing diverse landscape features and climatic gradients. By integrating spatially explicit landscape and climatic data with genomic information, we can gain deeper insights into how populations respond to different climatic pressures and identify genomic signatures of local adaptation (Dauphin et al. 2023). Furthermore, integrating landscape genomics and eco‐evolutionary modelling enables prediction of ‘genomic offset’—the degree of genetic change required for a population to adapt under future climatic conditions (Rellstab et al. 2021; Chen et al. 2022). Such an integrative framework allows for more accurate identification of vulnerable populations and landscapes, thereby strengthening the development of targeted conservation strategies (Bay et al. 2018; Turbek et al. 2023).

Such an integrative framework is particularly critical in the Sino‐Himalayan region—a tectonic collision zone between the Indian and Eurasian plates that gave rise to the Himalayas and the Qinghai–Tibetan Plateau (QTP) over the past 50 million years. The region's topographic complexity, including extensive mountain chains, deep valleys, and high plateaus, has created strong biogeographic barriers that promote isolation, diversification, and a high level of endemism (Favre et al. 2015). As a global biodiversity hotspot, the Sino‐Himalayan region supports numerous species adapted to some of the world's most extreme environments, making it a suitable setting for studying the interplay between geography, climate and evolutionary processes. Previous research in the Sino‐Himalayan region has shown that geological uplift during the Pliocene, followed by repeated glacial–interglacial cycles, played a major role in shaping species divergence across the Qinghai–Tibetan Plateau and its surrounding mountain systems (An et al. 2009; Favre et al. 2015; Yao et al. 2022; Jiao et al. 2024; Wei et al. 2025). Recent studies have identified geographic barriers and signals of local climatic adaptation in birds, mammals and plants from this region (Dahal et al. 2017; Jia et al. 2020; Chen et al. 2022, 2023; Sang et al. 2022; Tang et al. 2025; Zhang et al. 2025). However, studies that jointly examine both geographic and environmental heterogeneity remain limited in number and scope and often are only focused on a narrow portion of species' ranges or on taxa with similar life‐history traits. Consequently, a comprehensive understanding of how alpine species persist across their full geographic distributions—particularly in regions where steep topographic barriers coincide with sharp climatic gradients—remains lacking.

**FIGURE 1:**
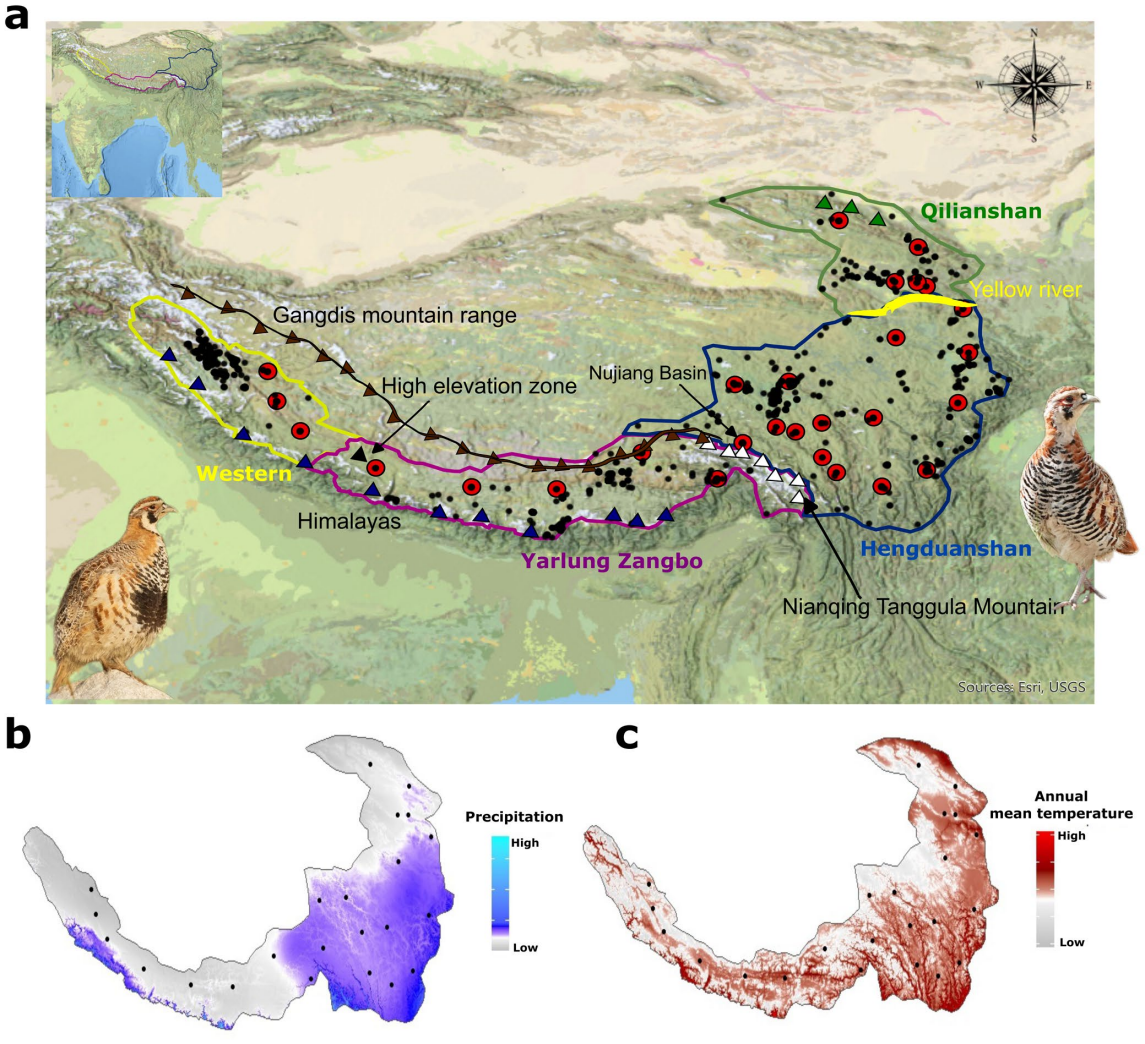
Distribution range of the Tibetan Partridge and environmental variability across the Sino‐Himalayan Landscape. (a) The map inset (top left) illustrates the Indian subcontinent for geographical reference. The main panel highlights the distribution range of the Tibetan Partridge, along with key mountains and geographical barriers. Sampling locations are represented by red circumpuncts, while black dots indicate known distribution points based on the eBird database. The high‐elevation zone along the upper Yarlung Zangbo River is marked with a black triangle, and prominent mountain ranges include, the Himalayas (blue triangle), Gangdis Mountains (brown triangle with connecting brown line) and Nianqing Tanggula Mountains (white triangle), Qilian Mountains (green triangle). The Yellow River is depicted in yellow. The Tibetan Partridge populations west of the Nianqing Tanggula Mountains are noted for their distinctive black belly patch (bottom left image, Neeraja V, ML621931282), contrasting with eastern populations that lack this feature (bottom right image, Nick Addey, ML612172194) (b) Annual precipitation (mm) and (c) annual mean temperature (°C) maps highlight climatic variability across the distribution range.

To address this knowledge gap, we have utilised the Tibetan Partridge ( *Perdix hodgsoniae* ), a high‐altitude endemic bird inhabiting montane shrublands between 2800 and 4600 m (McGowan and Madge 2010) in the Sino‐Himalayan region (Figure 1a). This species is particularly well suited for studying alpine adaptation because its dispersal is extremely limited; *P. hodgsoniae* relies primarily on walking, which promotes the formation of multiple localised populations across their range (Lu and Ciren 2002). Prior work on *P. hodgsoniae* has identified physiological and genetic mechanisms, including the *ESR1* gene and gene‐expression plasticity, that contribute to high‐altitude adaptation (Palacios et al. 2023; Wang et al. 2025). This positions the species as a model for understanding how alpine‐specific environmental challenges translate into genomic adaptation, with broader relevance for other alpine birds with limited dispersal.

Beyond its biological attributes, the spatial complexity of the Tibetan Partridge's range provides a compelling natural setting to investigate the interplay between geographic barriers, climatic gradients and population divergence. The western portion of its range—bounded by the Himalayas to the south, the Gangdis Mountains to the north and the Nianqing Tanggula Mountains in the central east—experiences lower precipitation (Favre et al. 2015) (Figure 1a,b). In contrast, areas east of the Nianqing Tanggula Mountains, characterised by mid‐elevation valleys, receive higher precipitation (Figure 1a,b). The species' northern distribution is limited by the Qilian Mountains, with the Yellow River and its tributary, the Huangshui River, forming a natural boundary that separates it from the Hengduan Mountains (Figure 1a). This combination of rugged topography and climatic gradients creates a mosaic of isolated microhabitats, restricts dispersal and imposes region‐specific selective pressures, providing a natural experiment for testing how topography × climate interactions drive population divergence.

Given the fragmented and heterogeneous landscape of the Tibetan Partridge's range, we hypothesise that both geographic and environmental factors jointly shape its population divergence. Precipitation varies more dramatically than temperature across the species' range—especially across major mountain barriers (Figure 1b,c) and this east–west precipitation gradient is well documented to influence species richness in birds and small mammals in this region (Ernst 2004; Price et al. 2011; Srinivasan et al. 2014). Guided by this ecological context, we further hypothesise that precipitation represents a particularly important ecological driver of local adaptation in Tibetan Partridge populations. Building on these ideas, we articulate mechanistic hypotheses grounded in landscape and evolutionary theory: (a) Major ridges, valleys and rivers restrict dispersal and create topographic barriers to gene flow resulting in spatially structured populations (b) Geographic barriers interact with climatic gradients to generate region‐specific selective pressures that drive local adaptation and (c) The combined effects of topography and environmental heterogeneity result in spatially variable adaptation, such that some populations may be more vulnerable than others under future climate change.

This study provides a conceptual and methodological advance by integrating mechanistic hypotheses, range‐wide sampling and population‐genomic analyses to investigate how topography and climatic heterogeneity interact to drive population divergence and local adaptation in alpine birds. By integrating range‐wide genomic data, landscape and climatic analyses and predictive eco‐evolutionary modelling, we capture fine‐scale population genetic structure across species' full distribution range. Integrating these data with eco‐evolutionary modelling allows us to identify populations most vulnerable to future climate change, highlighting potential evolutionary refugia and providing a framework applicable to the studies of other high‐altitude species facing rapid environmental change.

## 2 Materials and Methods

### 2.1 Sample Collection and Genome Re‐Sequencing

We employed an a priori comparative sampling strategy intended to capture (i) the full distribution of *P. hodgsoniae* and (ii) the most pronounced environmental heterogeneity across its range. Populations in the western and northeastern portions of the range are separated by major mountain barriers and experience markedly different precipitation regimes. This contrast provided a natural experimental framework for assessing the combined effects of topographic isolation and climatic gradients on genomic divergence.

Tibetan Partridges were captured following protocols designed to minimise stress and disturbance to the birds. Because this species is resident, group‐living and typically follows fixed daily movement paths (McGowan and Madge 2010), we first conducted field surveys to locate active groups and identify their regular travel routes within suitable habitats across the Qinghai–Tibet Plateau. Based on these observations, traps were placed along established movement paths in locations that allowed continuous monitoring from a distance to avoid disturbing the birds' natural behaviour. When a bird entered a trap, it was immediately removed to reduce handling time. We collected 0.5–1 mL of blood (approximately 0.14%–0.28% of body weight) from the brachial (wing) vein using a sterile 1 mL syringe, a sampling volume well within accepted ethical guidelines for wild birds. Blood samples were collected from 96 individuals of Tibetan Partridge across 28 different locations within the Qinghai–Tibetan Plateau (QTP), Sino‐Himalayan landscape (Figure 1a). Eight of these populations are located to the west of the Yarlung Zangbo river and associated mountain ranges, while 20 are from the north‐east regions, ensuring uniform sampling coverage across the distribution range of Tibetan Partridges. Blood samples were transferred to sterile tubes, stored in a portable cooler during fieldwork and subsequently transported to the laboratory, where they were frozen at −20°C until DNA extraction. Following sampling, each bird was released at its original capture location and observed to ensure normal recovery and behaviour.

Genomic DNA was extracted from samples using Qiagen's DNeasy Blood and Tissue kit (Qiagen, Valencia, CA, USA). Whole‐genome re‐sequencing was performed for each individual using the Illumina NovaSeq 6000 platform, and the raw sequencing reads were mapped against the previously published genome of Tibetan Partridge (Palacios et al. 2023) using BWA v0.7.17 with default settings (Li and Durbin 2009). Quality control of the mapped reads was conducted using PICARD (http://picard.sourceforge.net/) to remove PCR duplicates, followed by the Genome Analysis Toolkit (GATK) v.4.2.0.0 (McKenna et al. 2010) and GATK best practices recommendations (Van der Auwera et al. 2013) to perform base quality recalibrations, insertion/deletion (INDEL) realignment, Single Nucleotide Polymorphism (SNP) discovery, and genotyping across all 96 samples. The variant filtering was done using a pipeline previously developed in the lab (Lamichhaney et al. 2015), applying the following filtering parameters: Fisher strand bias > 15, mapping quality < 50, mapping quality rank sum < −0.1, read position rank sum < −3, base quality rank sum < −5, depth > 4000 and < 500, quality by depth < 5, SNP quality > 100 and genotype quality > 10. Variants genotyped across all 96 samples were retained, and the final VCF file was generated using VCFTools v.0.1.13 (Danecek et al. 2011) for downstream analyses.

### 2.2 Morphological Data Collection

Standard morphological measurements—including body mass, tarsus, wing, tail and bill metrics—were recorded rapidly to further limit handling stress. We measured morphological traits, including body length, tail length and wing length, using a flexible ruler. Beak length, head length, beak width, tarsus length and middle toe claw length were measured with a vernier calliper, and body weight was recorded using an electronic scale. To account for body size variations, we adjusted the morphological data for body weight by dividing raw morphological values by body weight. These adjusted trait values were then used to assess differences along the landscape through boxplots, Analysis of Variance (ANOVA) and principal component analysis (PCA).

### 2.3 Population Genomics Analysis

We used a filtered set of 5,068,604 high‐quality SNPs to assess genetic diversity by computing population genetics parameters such as heterozygosity and inter‐population genetic diversity (*F*_ST_) using VCFtools v.0.1.13 (Danecek et al. 2011). To identify phylogenetic relationships between samples, we generated a maximum‐likelihood phylogeny in FastTree v.0.2.1 with recommended default parameters for nucleotide alignments (Price et al. 2010). We further used SNAPP (Bryant et al. 2012) to estimate divergence time between seven phylogenetic groups ‐ two outgroups: Daurian partridge and Grey Partridge, and individuals from six populations of Tibetan partridge across four different landscapes (Figure 1a), i. Western; ii. Yarlung Zangbo high elevation; iii. Yarlung Zangbo, 2900 m elevation; iv. Hengduanshan (Nujiang valley); v. Hengduanshan (Sichuan); and vi. Qilianshan, based on phylogenetic tree structure and known geographical barriers. We used Yarlung Zangbo's 2900 m population and two Hengduanshan populations (Sichuan and Nujiang) as separate populations to build the divergence timeline. We used the divergence time of genus *Perdix* (4.37 MYA) (http://www.timetree.org/) to constrain with 0 offsets and a standard deviation of 0.005 million years. We ran BEAST v.2.6.3 (Bouckaert et al. 2019) for 5 million generations, with sampling conducted every 250 generations; the initial 10% was discarded as burn‐in. We ran Tracer v.1.7.2 (Rambaut et al. 2018) to ensure convergence of all parameters (ESS > 200) and TreeAnnotator v.2.6.4 (Drummond and Rambaut 2007) to generate the maximum clade credibility tree. We also employed Estimated Effective Migration Surfaces (EEMS) analysis (Petkova et al. 2016) to map corridors and barriers to gene flow across these landscapes.

We also estimated ancestry using a maximum‐likelihood approach in Admixture v.1.3.0 (Alexander et al. 2009) and plotted the admixture patterns across the landscapes using Mapmixture v.0.1.0 (Jenkins 2024) in R. The optimal number of genetic ancestries was selected using a cross‐validation procedure with the *K*‐means method implemented in Admixture. To examine the pattern of isolation by distance (IBD) and isolation by environment (IBE), we performed the multiple regression for distance matrices (MRM) with 999 permutations using genetic distance (*F*_ST_), geographic distance (km), and Euclidean distance of selected environmental variables (BIO2, BIO8 and BIO9, BIO12, BIO15, see methods for Genotype‐Environment Association (GEA) analysis below for details) using the ecodist package (Lichstein 2007) in R.

### 2.4 Genotype–Environment Association (GEA) Analysis

Although broad geographic and climatic differences between regions were previously known, we used Gradient Forest analysis (Ellis et al. 2012) to evaluate the contribution of individual environmental variables. This procedure allowed us to rank precipitation‐ and temperature‐related metrics based on their predictive importance, identify ecologically relevant variables and reduce redundancy among highly correlated predictors. Due to computational limitations, we only used ~500,000 SNPs from the longest scaffold (Scaffold 738) for this analysis. We ranked the importance of all 19 bioclimatic variables available in the WorldClim database from this region (Fick and Hijmans 2017) using the gradientForest analysis (Ellis et al. 2012) and selected five key, weakly correlated variables (|*r*| < 0.6): two precipitation related variables: Annual Precipitation (BIO12), Precipitation Seasonality (BIO15) and three temperature related variables: Mean Diurnal Range (BIO2), Mean Temperature of Wettest Quarter (BIO8) and Mean Temperature of Driest Quarter (BIO9) (Figure S1, Table S1).

We employed a combination of two independent approaches to identify genomic regions associated with bioclimatic variables. First, we utilised the Latent Factor Mixed Model (LFMM), a univariate analysis method that detects associations between allele frequencies and climatic variables (Frichot et al. 2013). To account for population structure in the dataset, we incorporated four latent factors identified by admixture analysis. We conducted five independent Markov Chain Monte Carlo (MCMC) runs, with a burn‐in period of 5000 iterations followed by 10,000 iterations. The results from these five runs were averaged and adjusted for multiple testing, applying a false discovery rate (FDR) correction of 1% as the significance threshold to identify candidate SNPs. To further validate and ensure the accurate identification of candidate SNPs, we calculated the Fixation index (*F*_ST_) between two groups (five individuals each exposed to high and low values for each bioclimatic variable) using the candidate SNPs generated by LFMM alongside a comparable number of randomly selected SNPs across the genome.

Next, we employed Redundancy Analysis (RDA), a multivariate approach, to examine the relationship between multiple environmental variables and SNPs using the vegan package in R (Capblancq and Forester 2021; Oksanen et al. 2024). Genetic variations associated with the environment were considered significant if they had loadings in the tails of the distribution, specifically within a standard deviation limit of 3.5 along the first three RDA axes, as recommended in (Forester et al. 2018).

### 2.5 Genomic Offset Prediction

We predicted genomic offsets using climate associated SNPs found in both LFMM and RDA. Genomic offset estimates the mismatch between current and predicted future genotypes (Rellstab et al. 2021). Higher offsets indicate reduced adaptive capacity under future climates. We used a machine learning‐based non‐parametric modelling approach to predict allelic turnover under future climatic conditions, using the R package gradientForest (Ellis et al. 2012). This method calculates genetic offset as the Euclidean distance between genomic compositions in the current and predicted future climatic scenarios. To carry out this analysis, we downloaded three general circulation models (GCMs)—FIO‐ESM‐2, IPSL‐CM6A‐LR and UKESM1‐0‐LL, from the Worldclim database (http://www.worldclim.org) (Fick and Hijmans 2017). These models correspond to two carbon emission scenarios based on shared socioeconomic pathways (SSPs): SSP 2‐4.5 (moderate) and SSP 5‐8.5 (harsh), for two time periods: 2061–2080 and 2081–2100, all at a resolution of 30 s. For each given GCM, time period and SSPs, we built Gradient Forest (GF) models (12 models) and estimated the genetic offset for the same five bioclimatic variables previously used in Gene–Environment Association Analysis. We averaged the predictions for each SSP across the two time periods, yielding four overall predictions (SSPs 2‐4.5‐year 2070, SSPs 5‐8.5‐year 2070, SSPs 2‐4.5‐year 2090 and SSPs 5‐8.5‐year 2090). We used the Wilcoxon rank test to quantify the pairwise statistical differences of genomic offset between the four landscapes.

### 2.6 Niche Modelling

To assess the current climatic niche and predict future suitability for the Tibetan Partridge, we utilised occurrence data of Tibetan Partridge collected from field sampling and the GBIF database (GBIF.org 2023). After filtering occurrences at a 1‐km resolution, we retained a total of 230 data points, with 96 occurrences from the eastern range and 134 from the western range. We modelled these two ranges separately to identify bioclimatic variables that serve as significant predictors of niche suitability. We used the same bioclimatic data downloaded for genomic offset predictions used above. We further employed the USDM (v.2.1) package (Naimi et al. 2014) in R to assess the variance inflation factor (VIF) and identified multicollinearity among predictor variables, removing any variables with a VIF greater than 2.5. Additionally, we evaluated the variable importance using the Biomod2 package (Thuiller et al. 2016), retaining only those with higher variable importance. We randomly sampled 1000 background points for both the western and north‐eastern landscapes. We applied three iterations of the cross‐validation procedure, randomly dividing occurrence records into two subsets: 70% of the data for model calibration and the remaining 30% for testing. Model evaluation was based on the Receiver Operating Characteristic (ROC), a threshold‐independent indicator of model performance (Hanley and McNeil 1982), and the True Skill Statistic (TSS), which is calculated as the sum of sensitivity and specificity minus one (Allouche et al. 2006). The predictions from various modelling algorithms were summarised using a weighted mean approach (Marmion et al. 2009), excluding models with a TSS of 0.60 or lower in the final model construction.

We also quantified climatic niche divergence by comparing present climatic conditions with reconstructed Holocene (early, mid and late Holocene, downloaded from www.paleoclim.org at 2.5 min resolution, (Brown et al. 2018)) climatic periods using an ordination method described in (Broennimann et al. 2012). Both present and Holocene climatic niche datasets utilized 19 bioclimatic variables. All raster layers were standardised to a common resolution, projection and extent before extraction. To characterise the environmental background, 10,000 random points were sampled across the study area for each period, and climatic values for all variables were extracted at these locations to generate period‐specific background matrices. Climatic values corresponding to species occurrences were extracted using the same layers. Background and occurrence‐based matrices from all time frames were combined and subjected to principal component analysis using the ade4 package (Dray and Dufour 2007) with variables centered and scaled, and the first two principal components that captured most of the climatic variation were retained to define climatic niche space, after which smoothened kernel density of occurrence for two timescales were projected. Climatic niche surfaces were generated using ecospat.grid.clim.dyn with a grid resolution of 100 units in the two‐dimensional climatic space, producing smoothed density distributions representing climatic availability and climatic conditions occupied in each period. All analyses were performed using R v.4.4.3 (R Core Team 2025) in RStudio v.2025.9.2.418 (Posit Team 2025).

### 2.7 Landscape Resistance Surface Analysis

We employed a simplistic approach to map the resistance surface across the Tibetan Partridge's distribution range, drawing from the methodology of a previous study (Chen et al. 2022). To calculate the resistance surface, we first computed the average values of key landscape features (elevation, standard deviation of elevation and slope) using the current distribution of Tibetan Partridges as a reference. For each grid in the study area, we calculated the absolute difference between the value of each landscape feature and the average value of that feature based on the current distribution. A smaller difference indicated lower resistance, while a larger difference suggested greater resistance to movement. Next, we incorporated habitat suitability information into the resistance surface by subtracting the value of each grid from the highest suitability value, where a lower resultant value indicated lower resistance and vice‐versa. To make the resistance surface easier to visualise and interpret, we rescaled the average of these continuous landscape values and habitat suitability to a scale from 1 (lowest resistance) to 10 (highest resistance) (Chen et al. 2022).

## 3 Results

### 3.1 Morphology

**FIGURE 2:**
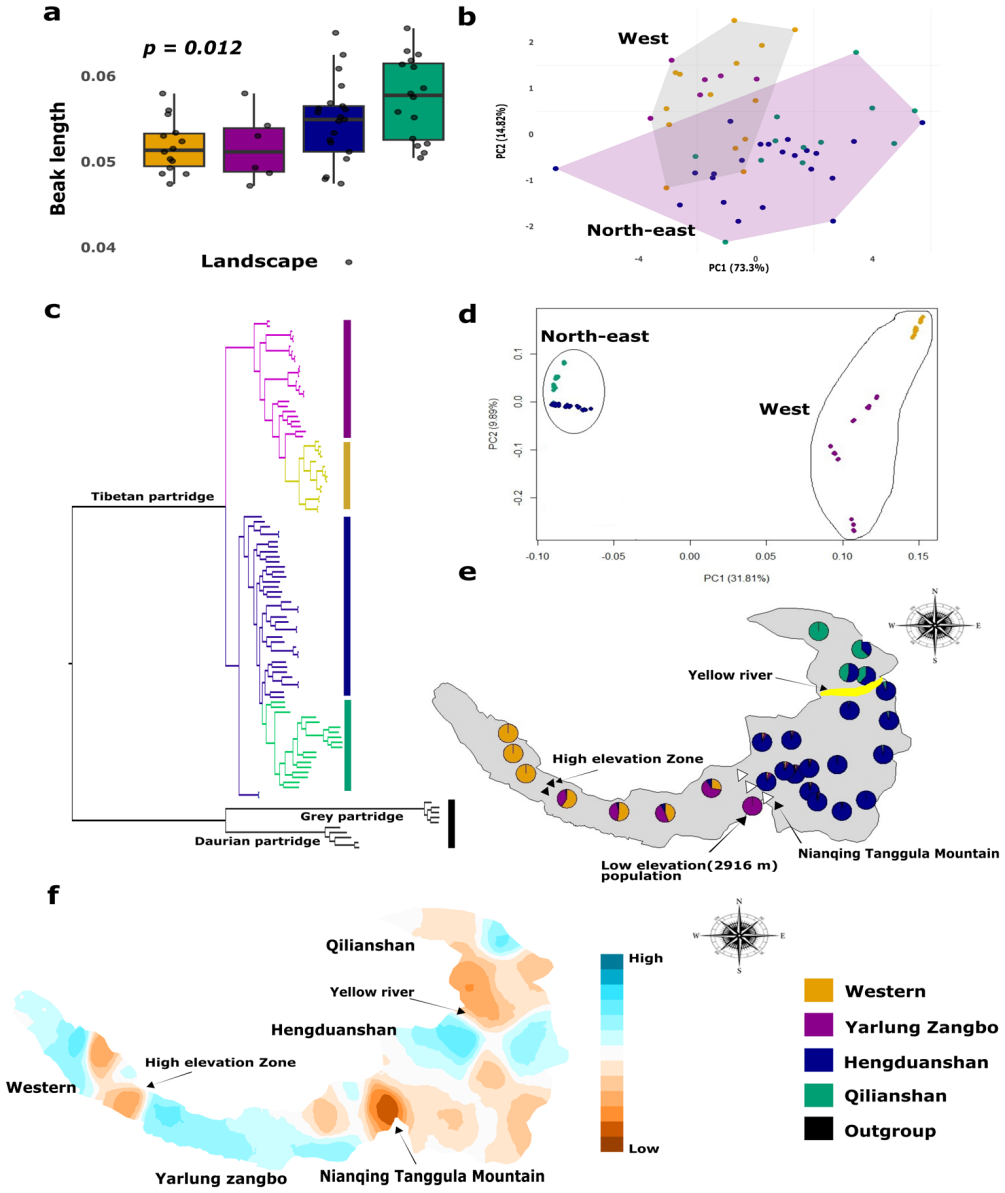
Morphological and genomic evidence reveal geographic and historical isolation as key drivers of genetic structure in Tibetan Partridges. (a) Beak length variation: Adjusted beak length (mm) by body weight across landscapes shows an increasing trend from the Western to the Qilianshan landscapes (b) Morphological PCA: Principal Component Analysis (PCA) of nine morphological traits indicates two distinct clusters, reflecting variation across Tibetan Partridge populations (c) Phylogenetic analysis: A maximum likelihood phylogenetic tree identifies the genetic structure of Tibetan Partridge populations, strongly aligned with their respective landscapes. All major nodes have local support > 0.9 based on the Shimodaira–Hasegawa test (Price et al. 2010) (d) Genetic PCA: PCA based on genomic data reveals clustering patterns within populations, consistent with the phylogenetic tree results, further validating the population structure (e) Admixture proportions map: A distribution map displays the admixture proportions for individuals assigned to each genetic cluster (*K* = 4). Key geographical features are annotated, including the high‐elevation zone along the Yarlung Zangbo River (black triangles), the Nianqing Tanggula Mountains (white triangles) and the Yellow River (yellow). This map was generated using the Mapmixture v.0.1.061 (Jenkins 2024) (f) EEMS Analysis: The Estimated Effective Migration Surface (EEMS) illustrates estimated migration rates with colour contours: Orange indicates low migration rates, while blue signifies high migration rates. Colour codes for each landscape are consistent across all panels for clarity and comparison.

We measured nine morphological traits in 60 individuals of Tibetan partridge across their distribution range in the Qinghai‐Tibet Plateau (QTP). Beak, foot and tarsometatarsus length were significantly different across landscapes (*p* < 0.05), with larger size in the north‐east populations (from Hengduanshan and Qilian landscapes), while wing and tail length showed little variation across the landscape but tended to increase from the western (Western and Yarlung Zangbo landscapes) to the northeastern regions (Figure 2a, Figure S2a–h). Principal component analysis (PCA) on these nine morphological traits clearly separated the western and north‐east populations (Figure 2b).

### 3.2 Genomic Structure and Historical Gene Flow

We further conducted whole‐genome sequencing of 96 individuals of Tibetan Partridges at ~20–25× average sequence coverage for each sample (Figure S3) from 28 different locations across these four landscapes (Figure 1a) and 10 individuals from two closely related species residing at lower elevations: Daurian Partridge ( *P. dauurica* ) and Grey Partridge ( *P. perdix* ). We used this genomic data to generate ~5.1 million high‐quality SNPs that were polymorphic in at least one population. A maximum likelihood phylogenetic tree based on these SNPs identified that populations from each of the four landscapes in western (Western and Yarlung Zangbo) and north‐east (Hengduanshan and Qilianshan) region formed genetically distinct clades (Figure 2c). Principal component analysis (PCA) using 291,338 Linkage‐Disequilibrium (LD)‐pruned SNPs revealed clustering patterns consistent with the phylogenetic tree, clearly separating populations from the western and north‐east landscapes (Figure 2d). Admixture analysis revealed distinct population structures at (a) both the western and north‐east edges of the distribution, (b) in the low‐elevation population (2900 m) within the Yarlung Zangbo landscape and (c) populations from the Hengduanshan landscape (Figure 2e). In contrast, other populations from the Yarlung Zangbo as well as those from Qilianshan displayed relatively high levels of admixture, suggesting greater gene flow among the populations within these regions. Consistent with these results, EEMS analysis identified the Nianqing Tanggula mountains, separating the habitats of western and north‐east populations, as a potential barrier to gene flow (Figure 2f). It also indicated that high elevation areas at the upper stretches of the Yarlung Zangbo region and north of the Yellow River could act as a barrier to gene flow (Figure 2f).

We estimated that the divergence of Tibetan partridge populations occurred between 0.33 and 0.05 million years ago (mya) (Figure S4a). The initial divergence took place between the western and north‐east populations around 0.33 mya, followed by the split of the Nujiang Basin population from the Qilianshan and Hengduanshan populations in the north‐east approximately 0.10 mya. A subsequent divergence occurred within a similar timeframe (~0.05 mya), separating the Qilianshan and Hengduanshan populations. Furthermore, Multiple Sequentially Markovian Coalescent (MSMC) analysis revealed similar demographic trajectories across populations, with a greater magnitude of increase in the northeast than the west. All populations show a post‐Pleistocene rise in effective population size, indicating range expansion and demographic recovery (Figure S4b).

Multiple Regression on Distance Matrices (MRM) analysis showed that environmental distance (*β* = 0.0771, *p* = 0.001) had a stronger effect on genetic differentiation than geographic distance (*β* = 0.0000433, *p* = 0.125). The full model explained 38.1% of the variation (*R* ^2^ = 0.381), confirming that environmental factors (IBE) play a greater role than isolation by distance (IBD) in shaping genetic diversity (Figure S4c,d).

### 3.3 Genotype–Environment Association (GEA) Analysis

**FIGURE 3:**
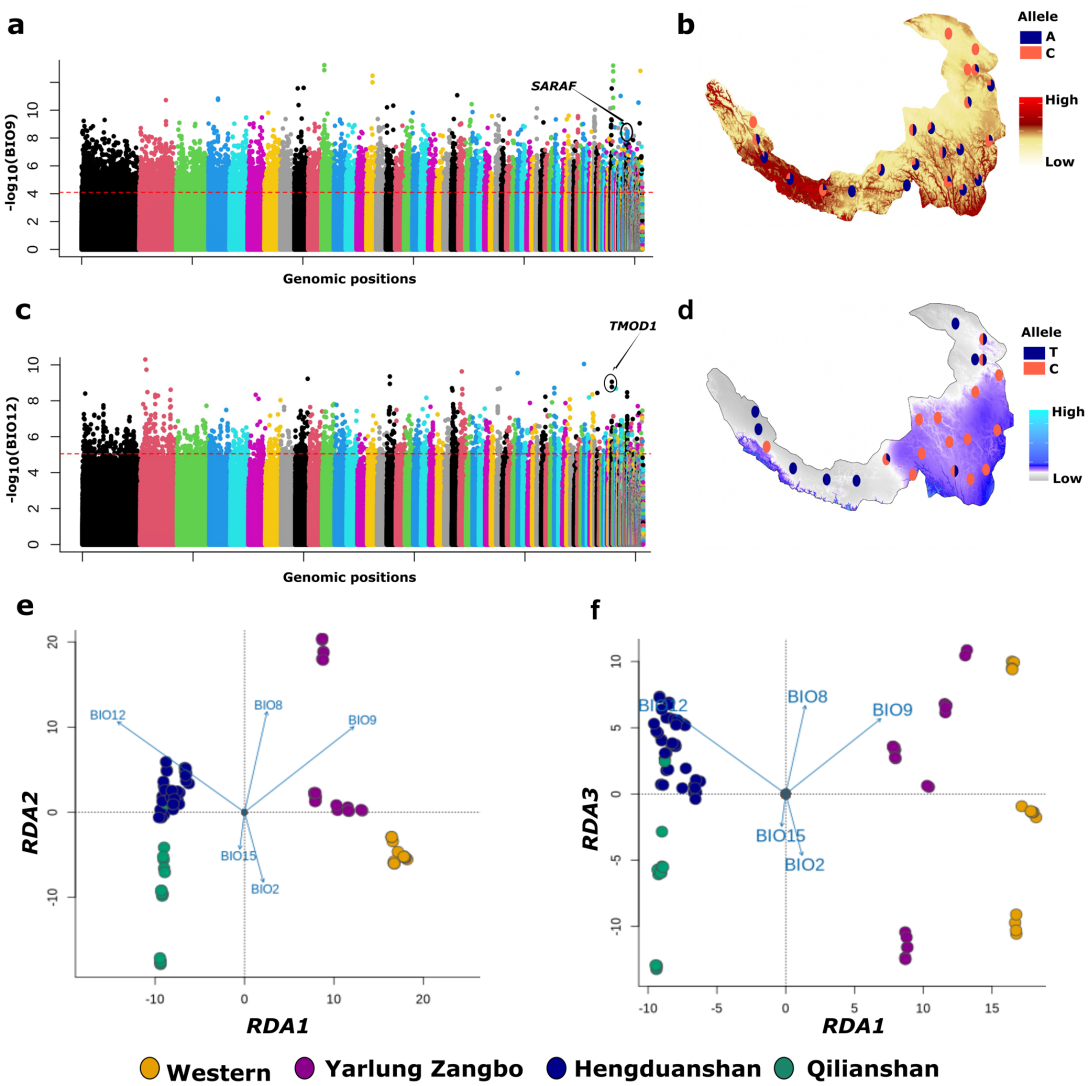
Genetic adaptations of Tibetan Partridge populations to local climatic conditions. (a) Manhattan plot showing −log_10_(*p*) values from the Gene–Environment Association (GEA) analysis for 5,068,604 SNPs with BIO9 (Mean Temperature of the Driest Quarter) as the environmental variable. Candidate SNPs surpassing the red dashed line meet the 1% False Discovery Rate (FDR) correction threshold. The *SARAF* gene (*Store‐operated calcium entry‐associated regulatory factor*), overlapping one of the top candidate SNPs, is highlighted within a circle (b) The allelic distribution of a candidate SNP (*Scaffold_631:373769*) within the *SARAF* gene demonstrates a higher frequency of the C allele in populations inhabiting regions with lower mean temperatures during the driest quarter (BIO9) (c) Manhattan plot showing −log_10_(*p*) values for SNPs associated with BIO12 (Annual Precipitation). Candidate SNPs surpassing the red dashed line meet the 1% False Discovery Rate (FDR) correction threshold. The *TMOD1* gene (*Tropomodulin‐1*), overlapping one of the top candidate SNP, is highlighted within a circle (d) The allelic distribution of a candidate SNP (*Scaffold_720:849107*) within the *TMOD1* gene reveals contrasting patterns: The T allele is nearly fixed in drier regions (white), while the C allele dominates in wetter regions (blue) (e) Redundancy analysis showing results of the first two axes (e) and first and third axis (f); Each dot represents an individual bird, colour‐coded by its population landscape (consistent with Figure 1). Blue vectors represent the direction and magnitude of correlation with environmental predictors represented by five composite bioclimatic variables principal component. Populations from Hengduanshan and Qilianshan landscapes are associated with precipitation‐related variables (Annual Precipitation: BIO12 and Precipitation Seasonality: BIO15), while populations from Western and Yarlung Zangbo landscapes are associated with temperature‐related variables (Mean Temperature of Driest Quarter: BIO9, Mean Temperature of Wettest Quarter: BIO8 and Mean Diurnal Range: BIO2).

LFMM analysis identified 442,926 candidate SNPs associated with these five bioclimatic variables after adjusting for multiple testing using FDR correction (*p* < 0.05) (Figure 3a,c; Figure S5a–c). Additionally, *F*_ST_ analysis confirmed that the candidate SNPs showed significantly greater genetic differences compared to an equal number of randomly selected SNPs (Figure S6a–e), suggesting true climate associations. Most (94.96%) of the candidate SNPs identified by LFMM analysis were associated with precipitation‐related variables (BIO12 and BIO15), whereas only relatively smaller proportions (5.04%) were associated with temperature‐related variables (BIO2, BIO8 and BIO9) (Figure S7).

Secondly, multivariate redundancy analysis (RDA) (Capblancq and Forester 2021) revealed consistent climatic associations. The first three RDA components explained, respectively, 66.76%, 17.47% and 6.18% of the variation. Individuals clustered by landscape, reflecting exposure to similar climates. Northeastern populations (Hengduanshan, Qilianshan) were associated with high precipitation (BIO12, BIO15), whereas western and Yarlung Zangbo populations correlated with temperature‐related variables (BIO2, BIO8, BIO9). These contrasting associations indicate precipitation‐driven adaptation in the northeast and temperature‐driven adaptation in drier western regions (Figure 3e,f).

We identified 14,999 SNPs associated with the five focal bioclimatic variables using RDA (at 3.5 SD), and 4377 of these overlapped with LFMM results (Figure S8). We refer to these overlapping SNPs as our ‘climate‐associated core candidate SNPs’ and used them for downstream analyses. Two illustrative examples include SNPs overlapping the *Store‐operated calcium entry‐associated regulatory factor* (*SARAF*) gene (Figure 3b, associated with BIO9), which influences heat‐stress responses through the mTOR1 pathway (Sanlialp et al. 2020), and *Tropomodulin‐1* (*TMOD1*) (Figure 3d, associated with BIO12), which plays roles in cytoskeletal stability and may mediate cellular responses to osmotic stress in contrasting precipitation regimes (Yamashiro et al. 2012). Most climate‐associated core candidate SNPs were non‐coding (*n* = 4313, 98.5%), and only 64 (1.5%) were coding SNPs (Table S2).

### 3.4 Genomic Offset and Niche Modelling

**FIGURE 4:**
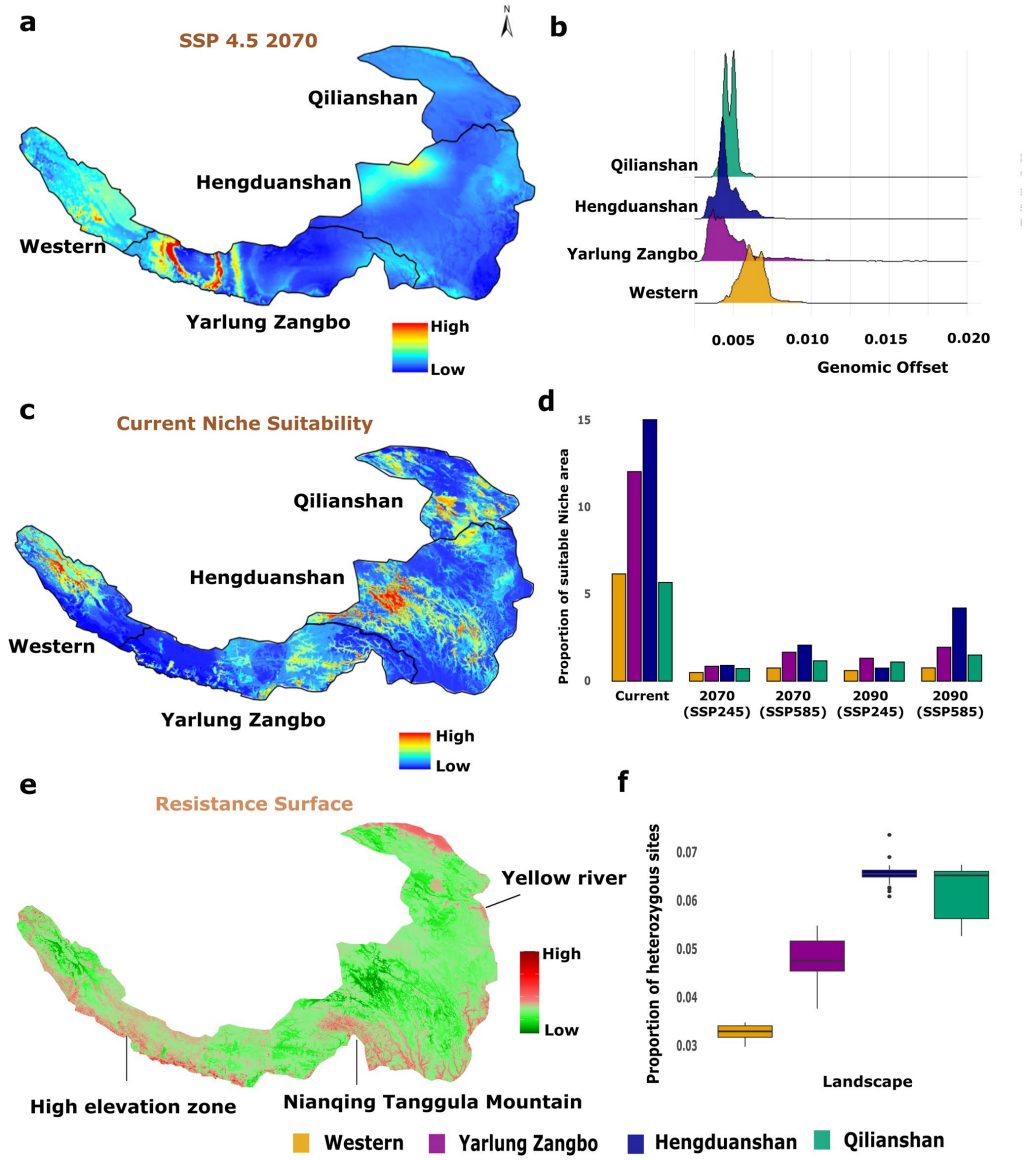
Climate change vulnerabilities in Tibetan Partridge populations revealed by landscape‐scale genomic offset and niche modelling across the Sino‐Himalayan region. (a) Genomic offsets predicted by the gradientForest model under SSP 2‐4.5 scenarios for 2061–2080 across the Tibetan Partridge distribution range (b) Genomic offsets are significantly higher in the Western (*n* = 267,215 points), Yarlung Zangbo (*n* = 564,031 points) and Qilianshan (*n* = 1,030,066 points) landscapes, whereas the Hengduanshan landscape (*n* = 317,063 points) shows the lowest genomic offset values (*p* < 0.0001 for all pairwise comparisons, two‐tailed Wilcoxon rank‐sum test with FDR correction) (c) Current niche suitability model projected across the Tibetan Partridge's distribution range (d) Proportions of suitable niche areas in each landscape under current and future climatic scenarios (SSP 2‐4.5 for 2070/2090 and SSP 5‐8.5 for 2070/2090) (e) Resistance surface map showing higher landscape connectivity (green) in the Hengduanshan and Qilianshan landscapes in the northeast compared to reduced connectivity in the Yarlung Zangbo and Western landscapes (f) Proportion of heterozygous sites in local Tibetan Partridge populations, indicating reduced heterozygosity in the Western landscape.

The heterogeneous Sino‐Himalayan landscape exposes Tibetan Partridge populations to divergent climatic regimes, resulting in region‐specific patterns of genetic variation and sensitivity to environmental change (Figure 1b,c; Figure S1a,b). As shown in the demographic modelling (Figure S4b), all populations experienced a shared post‐glacial increase in effective population size, but they differ in their recent Ne, with western populations showing comparatively smaller contemporary sizes. Building on this demographic context, we next evaluated whether these recently constrained populations are also those predicted to exhibit greater genomic offset under future climate change scenarios. Gradient Forest (GF) analysis predicted an increase in genomic offset over time (2070–2090) and under more extreme climatic scenarios (Figure 4a,b, Figure S8a–d). At the landscape scale, higher genomic offsets were predicted at the edges of the species' range, peaking in the dry western landscape, moderate in Qilianshan and Yarlung Zangbo and the lowest in the wetter Hengduanshan landscape (Figure 4a,b).

Climatic predictors of habitat suitability varied regionally: temperature and precipitation jointly shaped suitability in the west (BIO2, BIO4, BIO8, BIO15, BIO19), whereas precipitation‐related variables (BIO14, BIO15) dominated in the northeast (Table S3). Current niche predictions indicate only 38.9% of the species' range remains climatically suitable, concentrated mainly in the Hengduanshan region (15.1%) and Yarlung Zangbo (12%), and least in the western (6.1%) and Qilianshan (5.6%) regions (Figure 4c,d). Suitability declined sharply under future climate scenarios, especially in arid western landscapes (Figure 4d). Niche suitability comparison of Holocene and present climate showed complete divergence in the niche, depicting changes in the climatic niche of Tibetan partridges (Figure S9). Resistance surface analysis also revealed strong movement barriers across the Nianqing Tanggula Mountains (Figure 4e), consistent with EEMS results (Figure 2f), and higher connectivity within wetter northeastern regions. The calculation of the proportion of heterozygous sites across the genome showed low heterozygosity in west landscape, particularly in the western and higher in the northeast landscapes (Figure 4f).

## 4 Discussion

In this study, we integrated morphological, genomic, climatic, ecological and landscape data to investigate how a high‐altitude, low‐dispersal resident bird persists across one of the world's most topographically complex mountain systems. Our morphological and genomic analyses reveal pronounced population divergence across the heterogeneous Sino‐Himalayan landscape. Through genotype–environment associations, genomic offset modelling and regional niche projections, we show that Tibetan Partridge populations are finely adapted to their local climatic regimes, with precipitation emerging as the predominant environmental driver. These distinct adaptive trajectories—shaped by sharp climatic gradients, limited dispersal and rugged geographic barriers—result in uneven population vulnerabilities under future climate scenarios. Our findings also identify the Tibetan Partridge populations most at risk and demonstrate how mountain systems simultaneously generate and restrict biodiversity.

### 4.1 Geography and Historic Isolation Shape Morphology and Population Genetic Structure

Our analysis of morphological and genomic data of Tibetan partridge populations showed clear morphology and genetic differences across the landscape. The biogeographic history of the Tibetan Partridge has been shaped by three major geographic barriers, resulting in the formation of regionally distinct genetic lineages. These divergence events are unlikely to reflect tectonic uplift, as the Qinghai–Tibetan Plateau (QTP) had attained near‐modern elevation by the late Pliocene (Wang et al. 2014; Deng and Ding 2015). Instead, the estimated divergence times (0.35–0.05 mya) coincide with mid‐ to late‐Pleistocene climatic oscillations, when repeated glacial–interglacial cycles fragmented habitats across the plateau. The earliest split (~0.33 mya) likely reflects interglacial isolation, while the subsequent divergences during Marine Isotope Stage (MIS) 3 (0.06–0.03 mya) correspond to extensive glaciation and habitat contraction across the QTP (Owen et al. 2003; Cui et al. 2018). Geological records from the northern Hengduanshan Mountains also indicate that MIS 3 glaciations were more extensive than those of the Last Glacial Maximum (LGM), supporting the interpretation that extensive glaciation during this period caused severe habitat fragmentation and population isolation across the plateau (Xu et al. 2010). These events mirror diversification patterns reported in other Tibetan plateau endemics, including the Snow Partridge ( *Lerwa lerwa* ) (Yao et al. 2022), suggesting common climatic drivers of speciation and demographic changes. The post‐glacial increase in effective population size of Tibetan partridge inferred from our demographic analysis is in line with the known pattern of post‐glacial demographic expansion and range shifts following glacial retreats in QTP (Lei et al. 2014; Liang et al. 2017; Ding et al. 2020). Moreover, the stronger signal of isolation by environment (IBE) compared to isolation by distance (IBD) further underscores the role of climate in shaping population divergence in the Tibetan Partridge. The correspondence between our estimated divergence times and major Pleistocene glacial cycles provides a plausible—though inherently inferential—explanation for the emergence of the observed population genetic structure.

The Nianqing Tanggula Mountains appear to act as a primary biogeographic barrier separating western and northeastern populations, a pattern also reported for other Sino‐Himalayan species (Liu et al. 2018; Du et al. 2020; Rana et al. 2021). Although the Yellow River has been recognised as another important barrier for other Tibetan plateau species (Liang et al. 2018; Xu et al. 2018; Liu et al. 2022), our results reveal that high‐elevation zones in the upper Yarlung Zangbo region represent previously unrecognised barriers to dispersal. In this region, the tight coupling of steep topography with elevational vegetation belts restricts species to their preferred habitat zone, creating a narrow high‐elevation corridor that limits the movement of shrub‐dependent species while allowing uninterrupted dispersal only for alpine specialists. This elevation and habitat‐driven filtering effectively form an overlooked barrier separating populations across the upper basin. Earlier phylogeographic studies, constrained by limited sampling across the Tibetan plateau, provided only a partial view of gene flow and population connectivity (Du et al. 2020; Chen et al. 2022, 2023; Jiao et al. 2024). In contrast, our extensive sampling across previously underrepresented regions provides a detailed assessment of how topography, glacial history and environmental gradients collectively influence genetic connectivity.

#### 4.1.1 Genetic Adaptation to Local Climatic Conditions

Previously known considerable variation in temperature and precipitation across the Sino‐Himalayan region (Ernst 2004; Price et al. 2011; Srinivasan et al. 2014) (also Figure 1b,c) provided a strong basis for examining climatic adaptation in Tibetan Partridges. Our GEA and niche modelling analysis indicates distinct climatic associations among Tibetan Partridge populations across their distribution range, aligning with our hypothesis. While precipitation‐related variables predominantly drive genetic variation in the northeastern populations, temperature plays a more significant role in shaping genetic variation in the Yarlung Zangbo and western landscapes. In dry regions, species must adapt to extreme heat and limited water availability, making temperature regulation crucial for survival (Fuller et al. 2021). These results highlight how distinct environmental pressures across a topographically complex landscape generate divergent adaptive trajectories within a single species.

Although our analyses identify genomic regions and genes statistically associated with climatic variables, determining their precise molecular functions, causal variants and physiological consequences would require controlled functional genomics experiments (e.g., expression assays, regulatory analyses, or gene‐editing approaches) (Storz and Wheat 2010). Without such evidence, constructing detailed adaptive narratives would risk over‐interpretation. For this reason, we have intentionally limited speculation and focused instead on the population‐genomic patterns and environmental associations. Importantly, these findings establish a critical foundation for future experimental studies that can directly test the mechanistic roles of the candidate genes we have identified.

### 4.2 Differential Climate Vulnerability Across the Landscape

Genomic offset predictions indicate that western populations, which exhibit the lowest genetic diversity, also show the highest predicted genomic offset, reflecting limited evolutionary potential and a heightened risk of maladaptation under future climate change. In contrast, populations in the Qilianshan region display moderate genomic offsets combined with higher genetic diversity and greater connectivity, suggesting an enhanced capacity to adapt via gene flow or range shifts. The Yarlung Zangbo population, despite exhibiting low genetic diversity, maintains moderate habitat suitability and shows evidence of elevational niche expansion, consistent with localised adaptation to harsh environmental conditions. In contrast, the Hengduanshan region emerges as a climate refugium, characterised by high genetic diversity, low predicted genomic offset, high niche suitability and relatively extensive climatically suitable habitat. These contrasting patterns underscore how regional environmental and genomic factors interact to shape population‐specific vulnerability and adaptive potential.

Comparison with paleo‐climatic data reveals complete niche divergence since the Holocene, suggesting that long‐term climatic differentiation has fostered distinct adaptive regimes, which are likely to shape each population's response to future environmental change (Figure S9). However, these reconstructions should be interpreted with caution, as they implicitly assume that climatic preferences are inferred from present‐day distributions. However, this long‐term ecological separation is consistent with our GEA results: north‐east populations show signatures of precipitation‐driven adaptation, whereas western populations exhibit stronger associations with temperature extremes. Together, the paleo‐niche patterns and contemporary genomic signals indicate that sustained climatic differentiation has reinforced divergent adaptation across the species' range. As a result, each population's future response to climate change is likely influenced by these historical ecological trajectories: western populations, already inhabiting arid and thermally stressful environments with lower genetic diversity, exhibit the largest predicted genomic offsets, whereas northeastern populations appear more resilient due to higher genetic diversity, greater connectivity and long‐term association with wetter, more stable climates.

### 4.3 Similar Vulnerability Trends in Regional Alpine Species

Species exposed to similar environmental conditions may be exposed to similar vulnerability driven by future environmental change (Chen et al. 2023). We found similar patterns of climate‐driven vulnerability in other alpine taxa of the western Qinghai–Tibet Plateau, including the Green‐backed Tit ( *Parus monticolus* ), Plateau Pika ( *Ochotona curzoniae* ), White‐rumped Snowfinch ( *Onychostruthus taczanowskii* ) and Rufous‐necked Snowfinch ( *Pyrgilauda ruficollis* ), whose western populations already occupy comparatively warm environments and are projected to exceed their thermal tolerances under continued warming (Chen et al. 2022, 2023). When combined with strong dispersal barriers, these conditions create compounded vulnerability arising from both limited connectivity and high climatic exposure. This exemplifies the high‐altitude paradox: the topography that generates biodiversity can also constrain populations by restricting movement, threatening long‐term persistence under environmental change. These observations align with classic alpine dynamics, where mountain ranges act as ‘sky islands’, isolating populations on discrete high‐elevation habitats with limited opportunities for dispersal (Love et al. 2023). Such geographic isolation, together with steep climatic gradients, underlies topography × climate driven diversification processes that characterise the Sino‐Himalayan region (Wang et al. 2024).

### 4.4 Vulnerable Populations and Developing Key Connectivity Corridors

From a conservation perspective, our results identify specific regions where maintaining or restoring connectivity is likely to have the greatest impact. Three landscape zones emerge as priorities: (a) the western–upper Yarlung Zangbo corridor, where facilitating movement could partially alleviate isolation of high‐offset western populations; (b) low‐to mid‐elevation passes, west of the Nianqing Tanggula Mountains, representing the most plausible routes linking western and eastern populations; and (c) the eastern Hengduanshan foothills, which contain the largest area of persistent climatically suitable habitat and may function as a long‐term refugium.

Among these, the corridor through Jiali County is particularly important (Figure S10). This area hosts extensive shrub habitats suitable for Tibetan Partridge and has been documented as a region of hybridisation for two other pheasant species, the Blood Pheasant ( *Crossoptilon crossoptilon* ) and Tibetan Eared Pheasant ( *Crossoptilon harmani* ) (Lu and Zheng 2001). Therefore, efforts to conserve habitat across Jiali County would not only benefit Tibetan Partridge but also other Sino‐Himalayan fauna to aid genetic diversity in western populations. Conserving or restoring these corridors is crucial for sustaining gene flow and mitigating climate‐driven risk (Loss et al. 2011).

## 5 Conclusion

Our study illustrates how biogeographic barriers, climatic variability and landscape connectivity jointly shape the evolutionary trajectories of a low‐dispersal high‐altitude species. Using the Tibetan Partridge as a model, we show that mountain topography and climate jointly shape evolutionary processes, promoting population divergence through geographic isolation while enabling the persistence of distinct lineages within stable microclimatic refugia. These findings highlight that evolutionary resilience in alpine environments depends not solely on genetic diversity, but on its interaction with climatic stability and the permeability of the landscape. This is especially relevant in the Sino‐Himalayan region, one of the world's most species‐rich yet insufficiently studied mountain systems, where numerous climate refugia and isolated centers of endemism continue to persist (Sun et al. 2017). As global change pushes montane ecosystems toward unprecedented climatic conditions, pinpointing where genetic diversity is maintained and where it is most vulnerable will be essential for designing effective, future‐oriented conservation strategies (Sánchez‐Montes et al. 2018; Rellstab et al. 2021).

## Author Contributions

S.L. and N.W. designed the study. N.W. carried out fieldwork, collected the blood samples and measurement of data and quantified the ecology and biogeography of the Tibetan Partridge with contributions from C.Y., T.Z., S.Z. and Z.J. P.G. conducted the bioinformatics analyses under the supervision of S.L. P.C. and N.D. performed the niche modelling analyses. P.G. and S.L. wrote the manuscript, and all authors contributed to reviewing and editing the final draft.

## Funding

This work was supported by the National Institutes of Health (R15GM152940‐01).

## Disclosure

Code availability: Scripts and workflow for all analyses done in this paper are available on the GitHub page https://github.com/Prashantevo/Tibetanpatridgeeeb.

## Conflicts of Interest

The authors declare no conflicts of interest.

## Supporting information


**Data S1:** mec70274‐sup‐0001‐Supinfo.pdf.

## Data Availability

All short‐read whole‐genome sequencing data have been submitted to the National Center for Biotechnology Information (NCBI) under BioProject Accession number PRJNA1198335.
