# Supplementary material for: Biogeography and Climate Drive Population Divergence and Genomic Vulnerability in High Altitude Endemic Bird
Source: Mol Ecol. 2026 Feb 21;35(4):e70274. doi: 10.1111/mec.70274 (PMC12924089; doi:10.1111/mec.70274)
Supplement: Supplementary file 1 — Data S1: mec70274‐sup‐0001‐Supinfo.pdf. [file MEC-35-e70274-s001.pdf]

Supplementary Table 1: Bioclimatic variables, their ranked importance from Gradient Forest analysis and correlation. Correlation with ranked and selected variables is mentioned. Green colored cells are selected bioclimatic variables.

Ranked importance	Variable	Selection	Reason
BIO12 = Annual Precipitation	Precipitation	Yes	
BIO15 = Precipitation Seasonality (Coefficient of Variation)	Precipitation	Yes	
BIO9 = Mean Temperature of Driest Quarter	Temperature	Yes	
BIO18 = Precipitation of Warmest Quarter	Precipitation	No	correlated with BIO12
BIO16 = Precipitation of Wettest Quarter	Precipitation	No	correlated with BIO12
BIO2 = Mean Diurnal Range (Mean of monthly (max temp - min temp))	Temperature	Yes	
BIO5 = Max Temperature of Warmest Month	Temperature	No	correlated with BIO9
BIO13 = Precipitation of Wettest Month	Precipitation	No	correlated with BIO12
BIO17 = Precipitation of Driest Quarter	Precipitation	No	Correlated with BIO15
BIO6 = Min Temperature of Coldest Month	Temperature	No	Correlated with BIO9
BIO7 = Temperature Annual Range (BIO5-BIO6)	Temperature	No	Correlated with BIO12
BIO19 = Precipitation of Coldest Quarter	Precipitation	No	Correlated with BIO15
BIO10 = Mean Temperature of Warmest Quarter	Temperature	No	Correlated with BIO9
BIO4 = Temperature Seasonality (standard deviation ×100)	Temperature	No	Correlated with BIO9
BIO1 = Annual Mean Temperature	Temperature	No	Correlated with BIO15
BIO11 = Mean Temperature of Coldest Quarter	Temperature	No	Correlated with BIO12
BIO8 = Mean Temperature of Wettest Quarter	Temperature	Yes	
BIO3 = Isothermality (BIO2/BIO7) (×100)	Temperature	No	Correlated with BIO9
BIO14 = Precipitation of Driest Month	Precipitation	No	Correlated with BIO15

Supplementary Table 2: Annotation of 4377 core SNPs detected by both LFMM and RDA

Variants	Number of SNPS
**Non-coding**	**4313**
intergenic_region	2485
intron_variant	1115
3_prime_UTR_variant	32
5_prime_UTR_premature_start_codon_gain_variant	3
5_prime_UTR_variant	7
upstream_gene_variant	395
downstream_gene_variant	276
**Coding**	**64**
missense_variant	27
synonymous_variant	31
splice_region_variant	1
splice_region_variant&intron_variant	4
splice_region_variant&synonymous_variant	1

Supplementary Table 3: Selection of variables for niche modelling: (a) Variance inflation factor (VIF) of 2.5 was used as a threshold to avoid variables with high collinearity. Models with TSS higher than 0.6 were then selected and (b) variables were ranked based on their contribution to the model using permutation importance and lowest ranking variables were removed (grey colored cells) to reduce model overfitting .

(a)

West		East	
**Bioclimatic Variables**	**VIF**	**Bioclimatic Variables**	**VIF**
BIO2	1.087061	BIO 2	1.270523
BIO 4	1.120357	BIO 3	1.632404
BIO 8	1.227893	BIO 5	1.343072
BIO 15	1.595717	BIO 14	2.144265
BIO 19	1.517092	BIO 15	1.735536

(b):

West		East	
**Variable Name**	**Variable Importance**	**Variable Name**	**Variable Importance**
Bio 15	0.394121	Bio 5	0.564269
Bio 8	0.243943	Bio 15	0.543565
Bio 19	0.211322	Bio 14	0.284633
Bio 4	0.191222	Bio3	0.075001
Bio 2	0.102934	Bio 2	0.032169

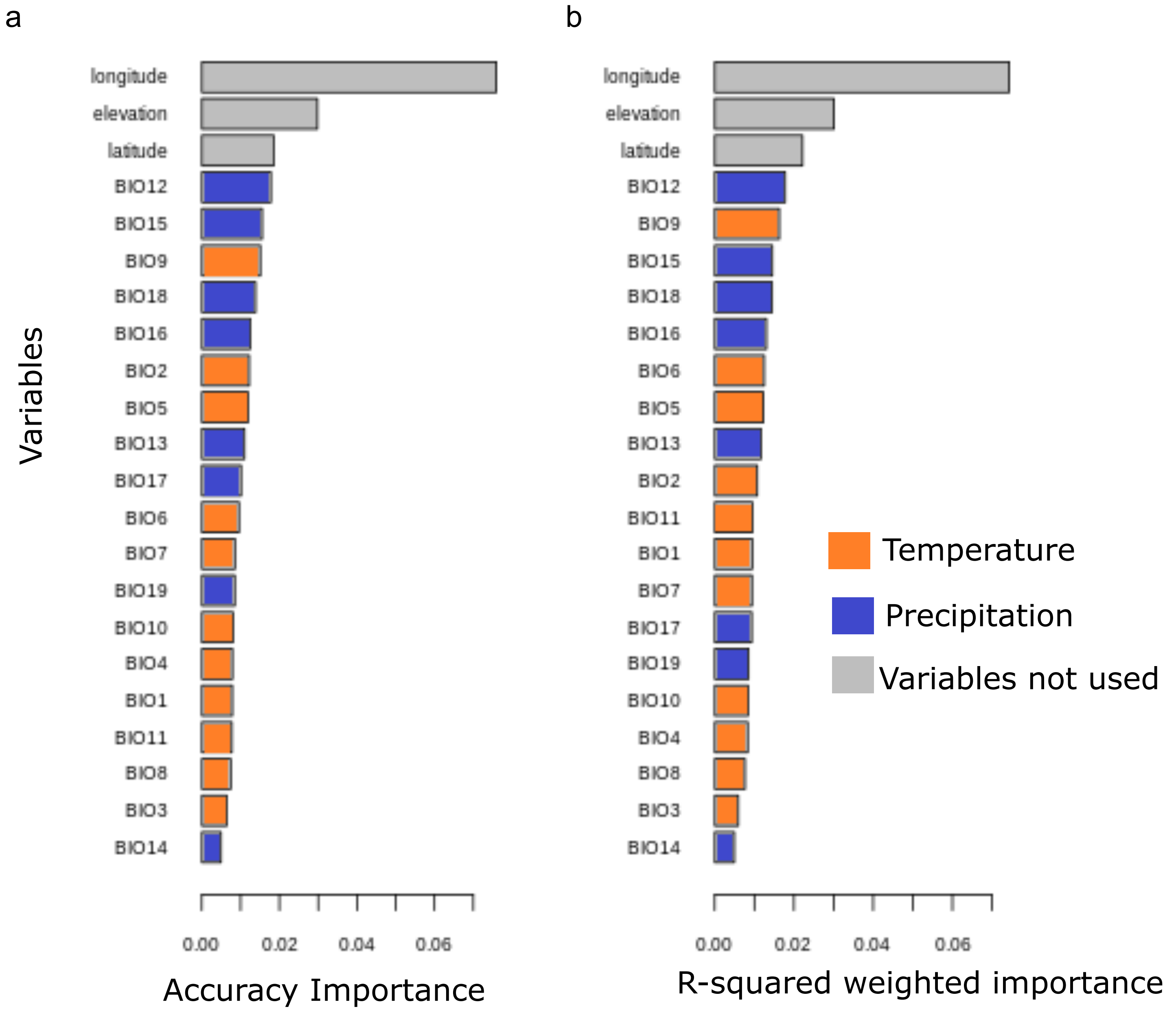

Supplementary Figure 1: Gradient forest analysis highlights precipitation-related variables (blue) as the strongest predictors of genetic variation in Tibetan Partridges (a) Ranked importance of variables based on prediction accuracy and (b) R-squared weighted importance reveals the dominance of precipitation-related factors. Variables in grey were excluded from further modeling to prioritize climate-related predictors.

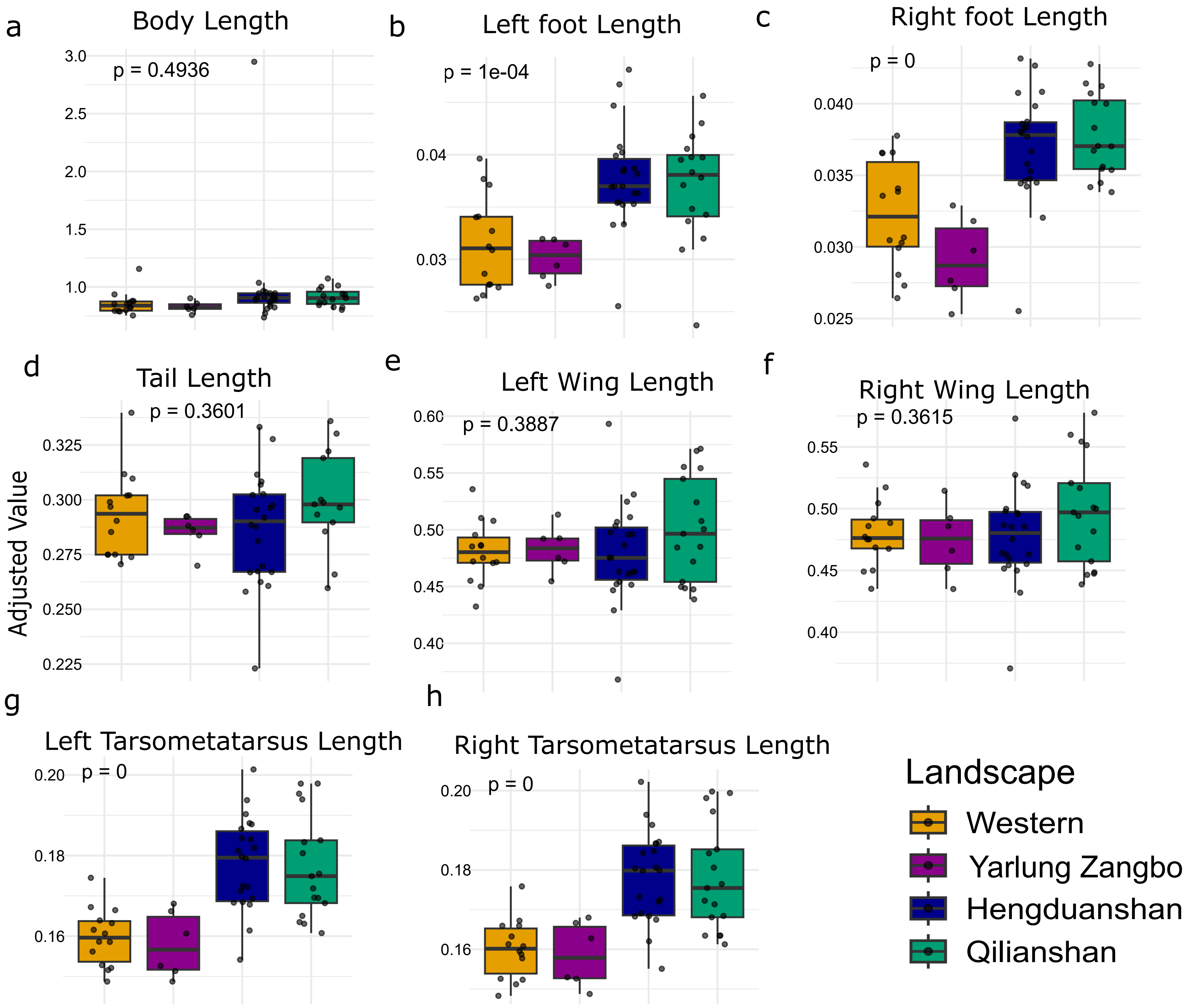

Supplementary Figure 2: Morphological trait variation among Tibetan Partridge populations residing across different landscapes (a) Body length, (b) Left foot length, (c) Right foot length, (d) Tail length, (e) Left-wing length, (f) Right-wing length, (g) Left tarsometatarsus length, and (h) Right tarsometatarsus length. P-values displayed are derived from Analysis of Variance (ANOVA), with $p < 0.05$ denoting significant differences in trait values across landscapes at the 95% confidence interval. Adjusted value for each trait calculated by dividing raw measure by body weight**.**

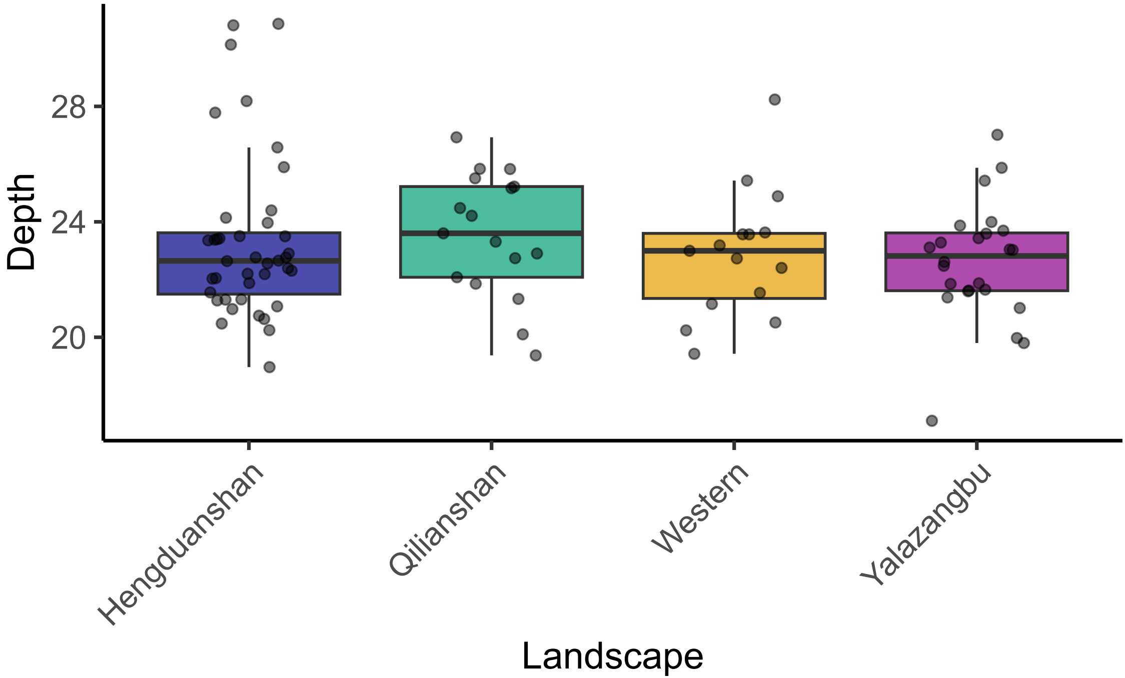

Supplementary Figure 3: Variation in sequencing coverage of individual samples across landscape. Gray dot referes to each individuals sample's coverage**.**

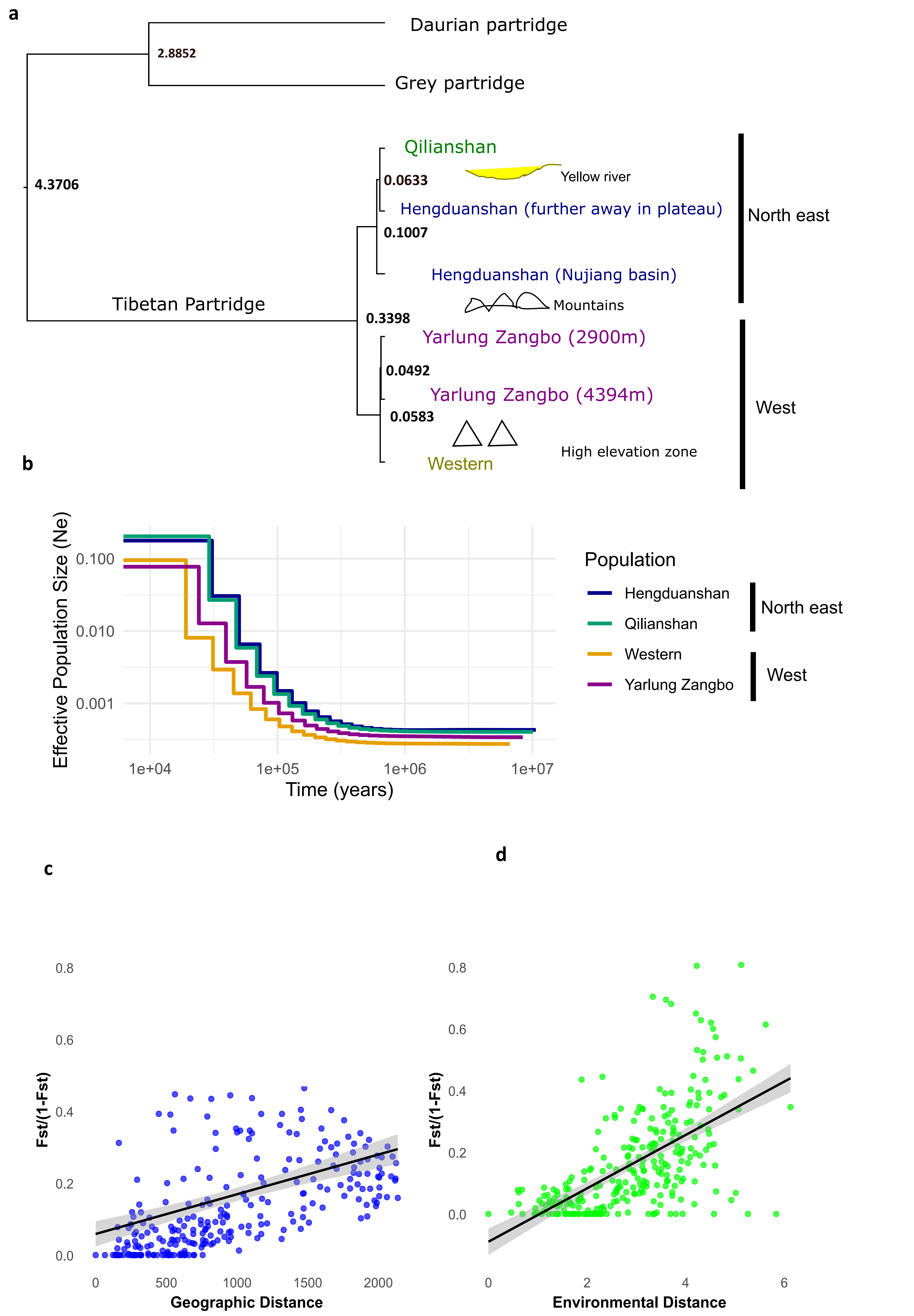

Supplementary Figure 4: Molecular dating and genetic divergence in Tibetan Partridge populations
(a) Molecular dating analysis indicates recent divergence within Tibetan partridge populations, with divergence times ranging from 0.33 to 0.05 million years ago (mya). The north-eastern and western populations, separated by the Nianqing Tanggula mountains, diverged around 0.33 mya. The population along the Nujiang river in Hengduanshan landscape, northeast of the Nianqing Tanggula mountains, diverged from the plateau population approximately 0.10 mya. The Yarlung Zangbo and Western populations, separated by high-elevation zones along the upper Yarlung Zangbo river, diverged around 0.05 mya; the most recent divergence within Tibetan partridges is low elevation movement to 2900 m. (b) MSMC-inferred demographic history of Tibetan Partridge populations across four landscapes on the Qinghai–Tibetan Plateau. Effective population size (Ne) is plotted against time. All populations show an overall increase in Ne toward the present, indicating post-glacial demographic expansion. Genetic variation across landscapes can be poorly explained by (c) Isolation by Distance (IBD), though its effects are relatively weak ($\beta = 0.0000433$, $p = 0.125$). In contrast, (d) Isolation by Environment (IBE) has a stronger and more significant influence ($\beta = 0.0771$, $p = 0.001$).

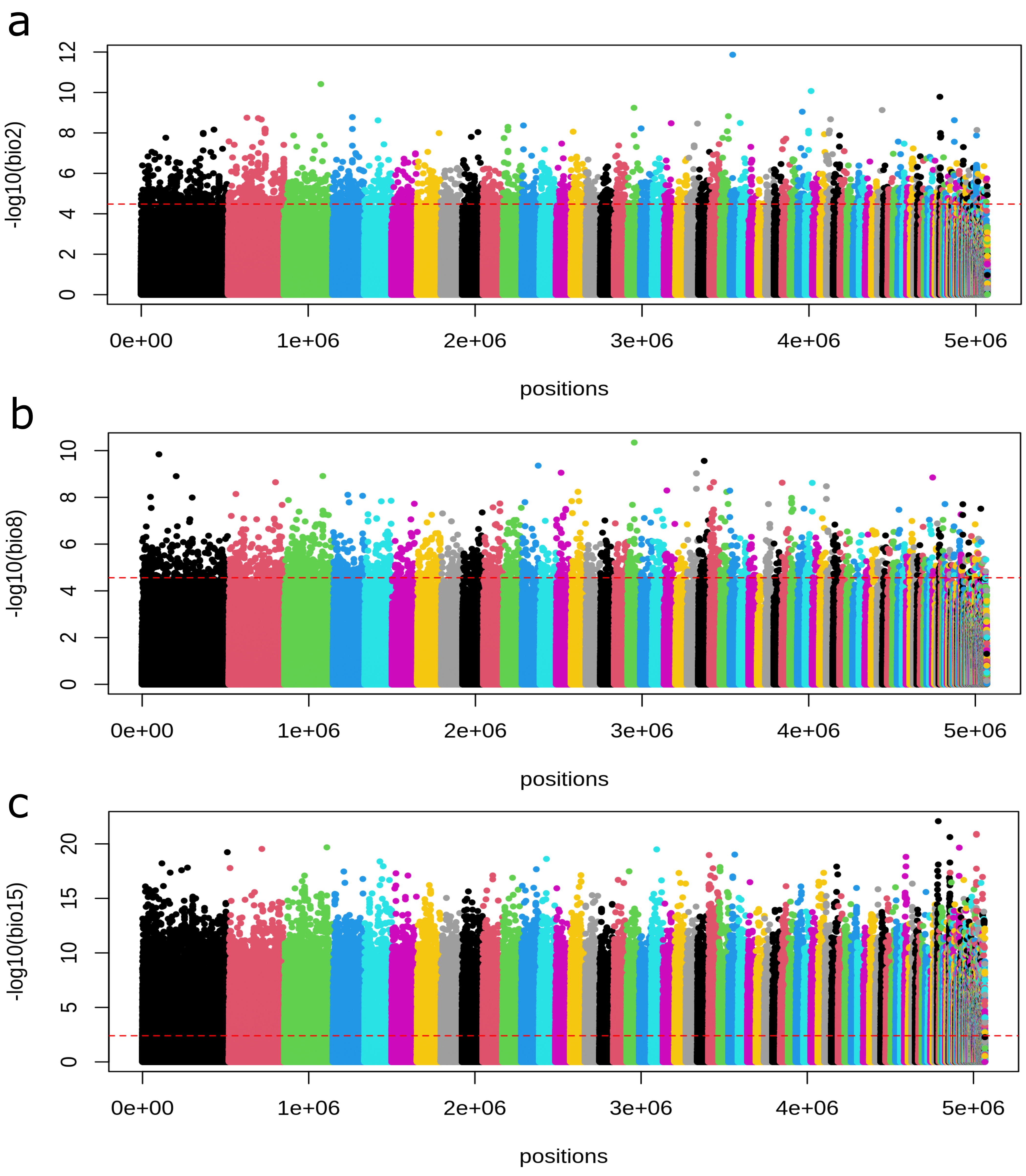

Supplementary Figure 5: Manhattan plot showing -log10(p) values from Gene Environment Association (GEA) analysis to identify association with bioclimatic variables (a) BIO2 (Mean Diurnal Range) (b) BIO8 (Mean Temperature of Wettest Quarter) and (c) BIO15 (Precipitation Seasonality). SNPs above the red dashed line denote candidate SNPs after a significance threshold of 1% False Discovery Rate (FDR) correction.

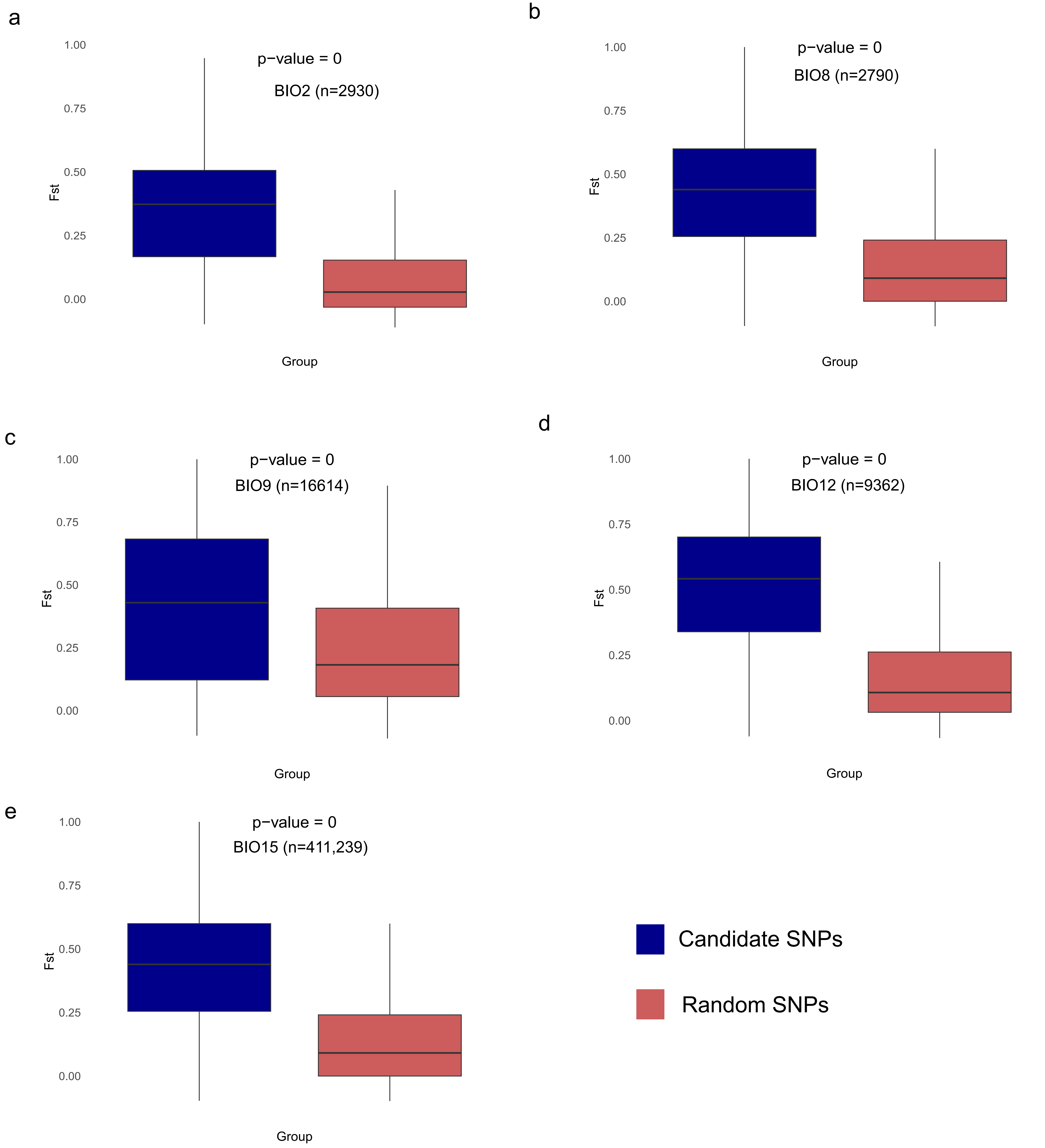

Supplementary Figure 6: Distribution of genetic differentiation (FST) for "climate-associated core" candidate SNPs vs. random SNPs, for (a) BIO2 (Mean Diurnal Range) (b) BIO8 (Mean Temperature of Wettest Quarter) (c) BIO9 (Mean Temperature of Driest Quarter) (d) BIO12 (Annual Precipitation) and (e) BIO15 (Precipitation Seasonality). FST calculations were based on ten individuals each from populations exposed to the highest and lowest values for each respective bioclimatic variable**.**

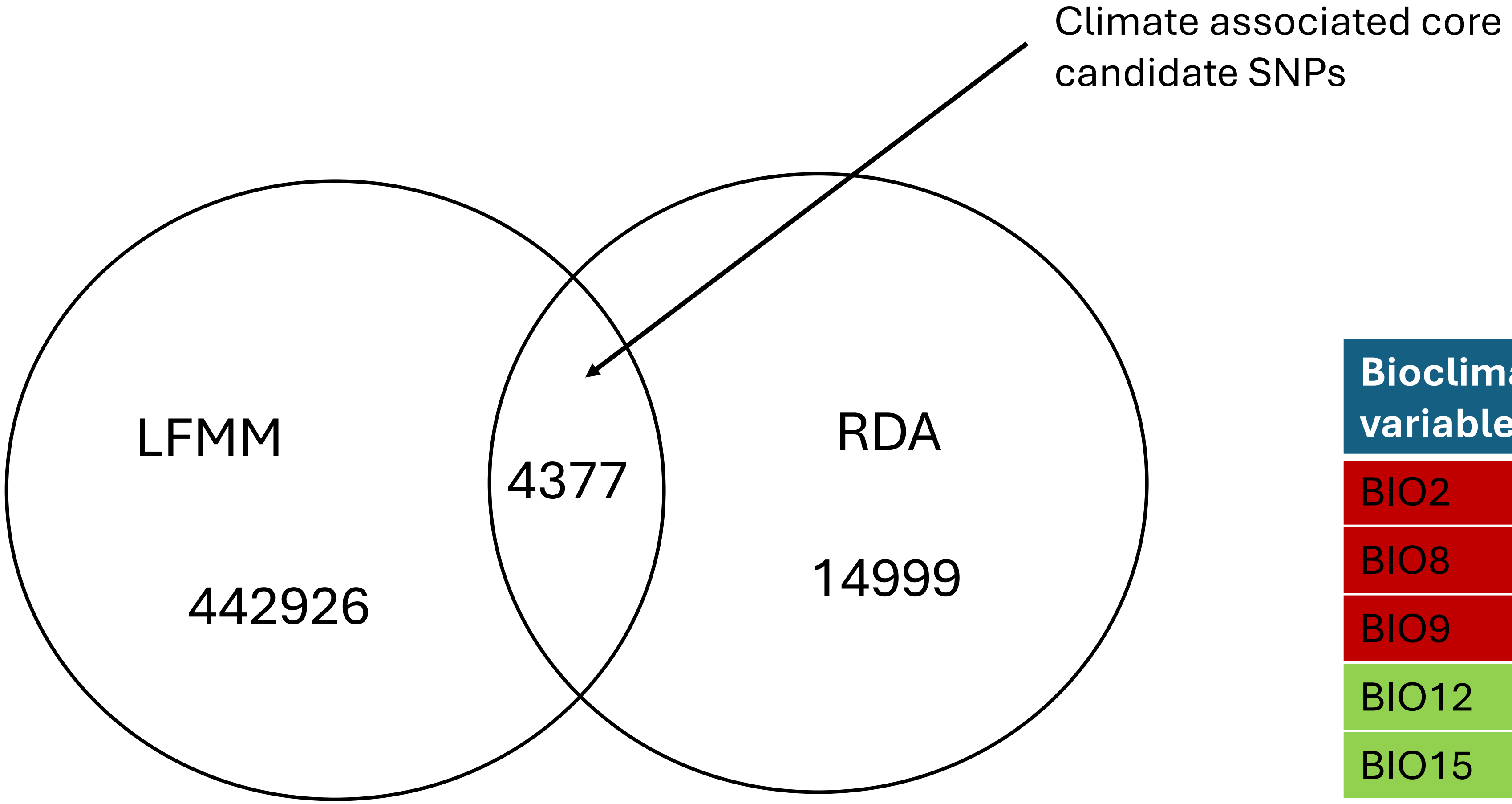

Bioclimatic variables	SNPs
BIO2	2930
BIO8	2790
BIO9	16614
BIO12	9362
BIO15	411230

Supplementary Figure 7: Venn diagram illustrating the overlap of candidate SNPs identified by univariate (LFMM) and multivariate (RDA) analyses associated with bioclimatic variables; center: overlapping SNPs identified by both LFMM and RDA, suggesting robust associations with climatic adaptation, lower left: number of SNPs identified by LFMM analysis for individual bioclimatic variables, with precipitation-related variables shown in green and temperature-related variables in red in the right table.

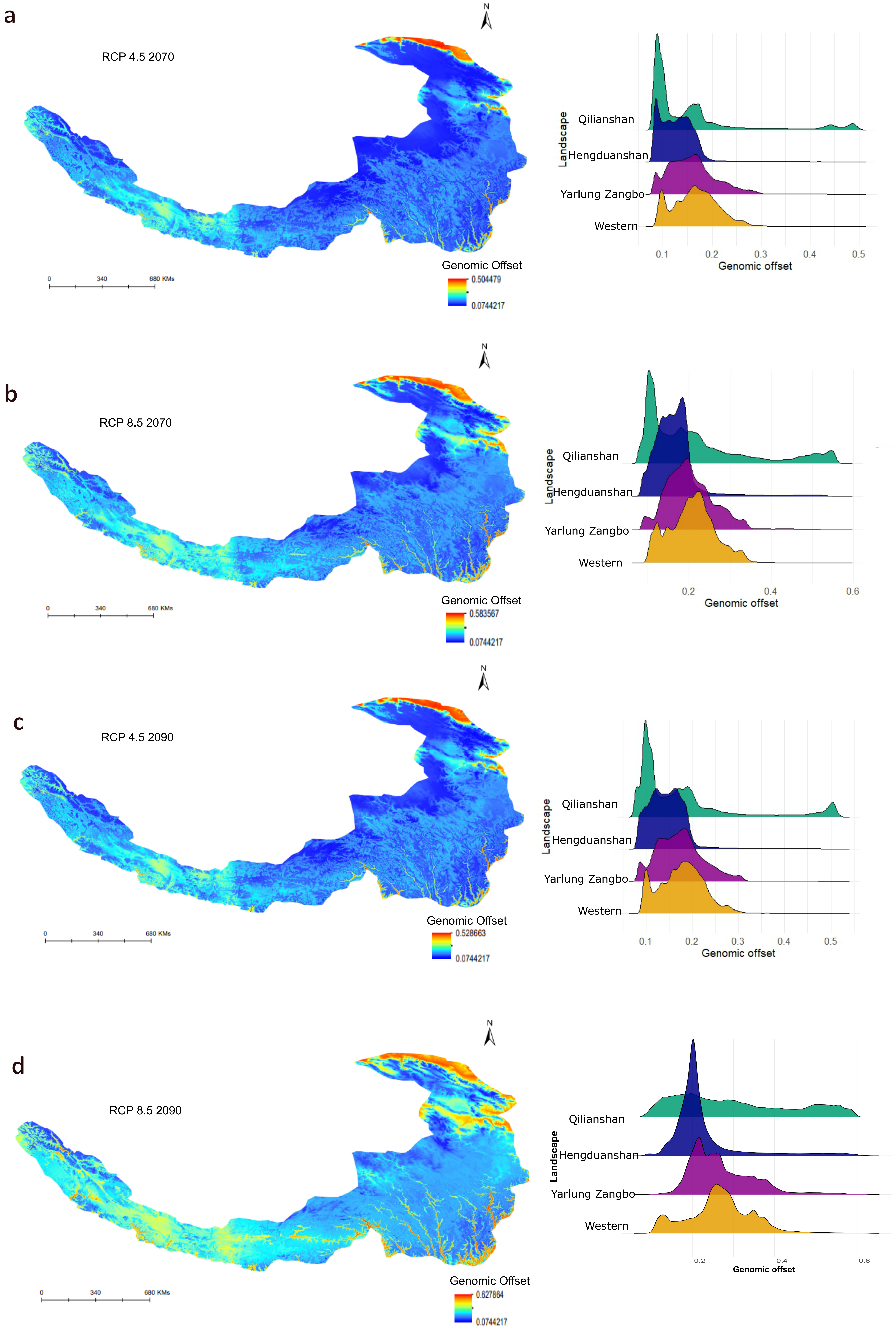

Supplementary Figure 8: The gradientForest predicted genomic offsets (a) under SSP 2-4.5 for the year 2070 ($p < 2e-16$ for all pairwise comparisons, the two-tailed Wilcoxon rank-sum test and FDR correction for multiple comparisons) (b) under SSP 5-8.5 for the year 2070 ($p < 2e-16$ for all pairwise comparison, the two-tailed Wilcoxon rank-sum test and FDR correction for multiple comparisons) (c) under SSP 2-4.5 for the year 2090 ($p < 2e-16$ for all pairwise comparison, the two-tailed Wilcoxon rank-sum test and FDR correction for multiple comparisons) (d) under SSP 5-8.5 for the year 2090 ($p < 2e-16$ for all pairwise comparison, the two-tailed Wilcoxon rank-sum test and FDR correction for multiple comparisons). SSP 5-8.5 is the worst climatic conditions and SSP 2-4.5 is moderate climatic conditions. The distribution of predicted genomic offset across each landscape is shown in the right panel for each figure.

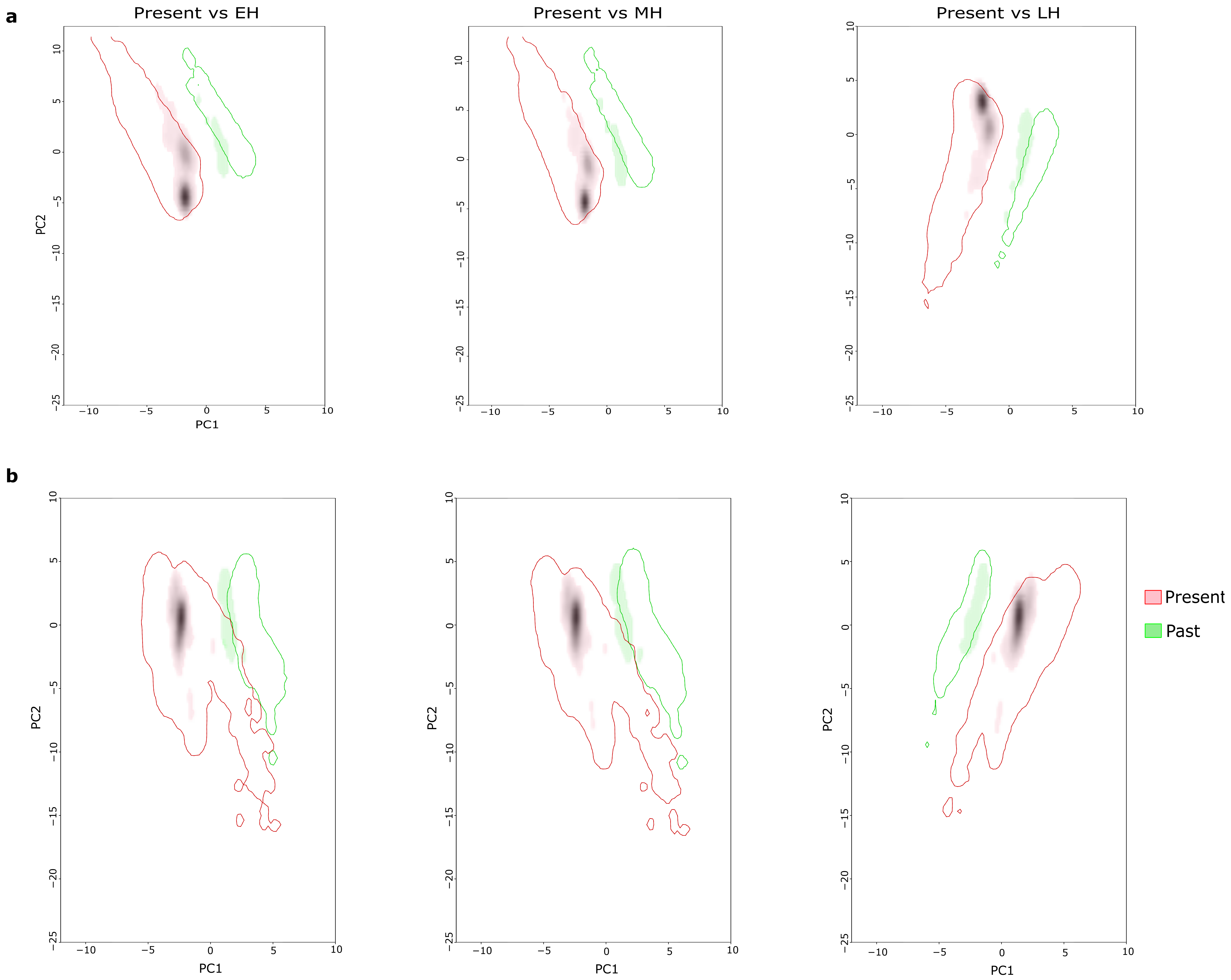

Supplementary Figure 9: PCA of niche modelling prediction of current and paleoclimate data shows complete niche divergence in Holocene-Present ; left panel - Present - Early holocene, middle panel - Present - mid-Holocene; right panel - Present - Late holocene in (a) West (b) North-eastern populations.

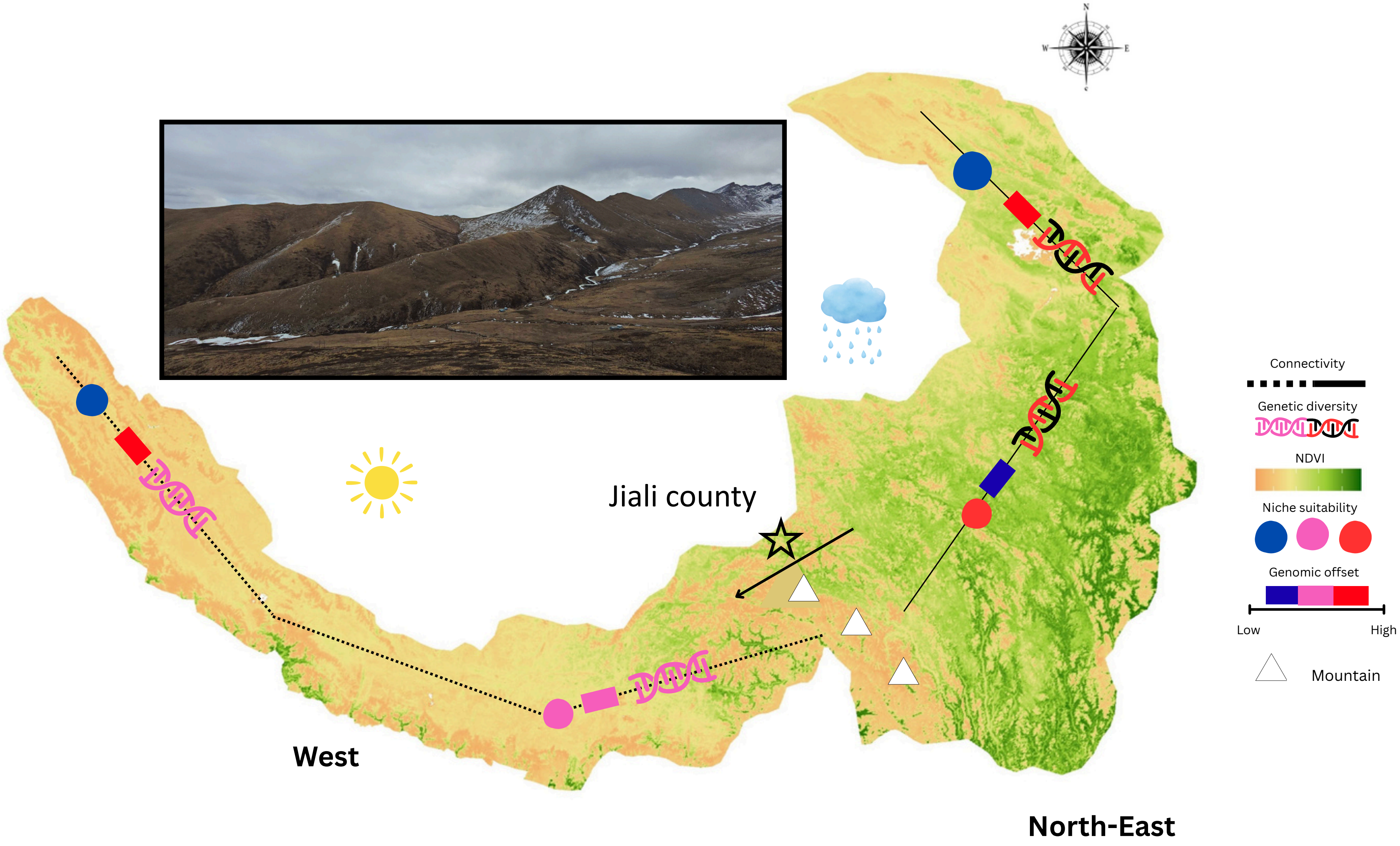

Supplemental Figure 10. Landscape-wide variation in habitat connectivity, genetic diversity, NDVI (Normalized Difference Vegetation Index), niche suitability, and genomic offset across the Sino-Himalayan distribution of the partridge.

The dry western portion of the range (sun icon) is characterized by low habitat connectivity, reduced genetic diversity, lower NDVI, poor niche suitability, and elevated genomic offset, indicating greater vulnerability to future climate change. In contrast, the wet northeastern region (rain icon) shows higher connectivity, greater genetic diversity, and more suitable habitat conditions. The arrow illustrates the inferred movement pathway from the northeast toward the west, facilitated through Jiali County (star), which appears to function as a key connectivity corridor. Inset: Representative landscape of Jiali County.
